# The PRECISE (PREgnancy Care Integrating translational Science, Everywhere) database: open-access data collection in maternal and newborn health

**DOI:** 10.1186/s12978-020-0873-8

**Published:** 2020-04-30

**Authors:** Laura A. Magee, Amber Strang, Larry Li, Domena Tu, Warancha Tumtaweetikul, Rachel Craik, Marina Daniele, Angela Koech Etyang, Umberto D’Alessandro, Ofordile Ogochukwu, Anna Roca, Esperança Sevene, Paulo Chin, Corssino Tchavana, Marleen Temmerman, Peter von Dadelszen, The PRECISE Network, Umberto D’Alessandro, Anna Roca, Hawanatu Jah, Ofordile Oguchukwu, Andrew Prentice, Melisa Martinez-Alvarez, Brahima Diallo, Adbul Sesey, Kodou Lette, Alpha Bah, Chilel Sanyang, Marleen Temmerman, Angela Koech Etyang, Peris Musitia, Mary Amondi, David Chege, Patricia Okiro, Geoffrey Omuse, Sikolia Wanyonyi, Esperança Sevene, Paulo Chin, Corssino Tchavana, Salesio Macuacua, Anifa Vala, Helena Boene, Lazaro Quimice, Sonia Maculuve, Eusebio Macete, Inacio Mandomando, Carla Carillho, Peter von Dadelszen, Laura A. Magee, Meriel Flint-O’Kane, Rachel Craik, Amber Strang, Marina Daniele, Donna Russell, Tatenda Makanga, Liberty Makacha, Yolisa Dube, Newton Nyapwere, Lucilla Poston, Jane Sandall, Rachel Tribe, Andrew Shennan, Sophie Moore, Tatiana Salisbury, Ben Barratt, Lucy Chappell, Sean Beevers, Kate Bramham, Aris Papageorgiou, Alison Noble, Hannah Blencowe, Veronique Filippi, Joy Lawn, Matt Silver, Matthew Chico, Judith Cartwright, Guy Whitley, Sanjeev Krishna, Marianne Vidler, Jing ( Larry) Li, Jeff Bone, Mai-Lei ( Maggie) Woo Kinshella, Beth A. Payne, Domena Tu, Warancha Tumtaweetikul, William Stones

**Affiliations:** 1 Department of Women and Children’s Health, School of Life Course Sciences, Faculty of Life Sciences and Medicine, King’s College London, Becket House, Room BH.05.11, 1 Lambeth Palace Road, London, SE1 7EU UK; 2 Department of Obstetrics & Gynaecology, Faculty of Medicine, University of British Columbia, Vancouver, Canada; 3 Centre of Excellence in Women & Child Health, East Africa, Aga Khan University, Nairobi, Kenya; 4 Medical Research Council Unit The Gambia at the London School of Hygiene and Tropical Medicine, Fajara, The Gambia; 5 Department of Physiological Science, Clinical Pharmacology, Faculty of Medicine, Universidade Eduardo Mondlane, Maputo, Mozambique; 6 Centro de Investigação em Saúde de Manhiça, Manhiça, Mozambique

**Keywords:** Open-source, Pregnancy, DHIS2, Placental disorders, eRegistry

## Abstract

In less-resourced settings, adverse pregnancy outcome rates are unacceptably high. To effect improvement, we need accurate epidemiological data about rates of death and morbidity, as well as social determinants of health and processes of care, and from each country (or region) to contextualise strategies. The PRECISE database is a unique core infrastructure of a generic, unified data collection platform. It is built on previous work in data harmonisation, outcome and data field standardisation, open-access software (District Health Information System 2 and the Baobab Laboratory Information Management System), and clinical research networks. The database contains globally-recommended indicators included in Health Management Information System recording and reporting forms. It comprises key outcomes (maternal and perinatal death), life-saving interventions (Human Immunodeficiency Virus testing, blood pressure measurement, iron therapy, uterotonic use after delivery, postpartum maternal assessment within 48 h of birth, and newborn resuscitation, immediate skin-to-skin contact, and immediate drying), and an additional 17 core administrative variables for the mother and babies. In addition, the database has a suite of additional modules for ‘deep phenotyping’ based on established tools. These include social determinants of health (including socioeconomic status, nutrition and the environment), maternal co-morbidities, mental health, violence against women and health systems. The database has the potential to enable future high-quality epidemiological research integrated with clinical care and discovery bioscience.

## Background

In less-resourced settings and particularly in sub-Saharan Africa, adverse pregnancy outcome rates are unacceptably high. This is reflected in global estimates of maternal mortality of 216/100,000 live births (2016 estimates), stillbirths of 18.4/1000 live births (2015 estimates), and neonatal mortality of 18.0/1000 live births (2017 estimates) [1–3]. While estimates of near-miss morbidity for mothers and newborns are thought to be eight-fold higher [4, 5], there is no widespread routine collection of relevant outcome data as there is for mortality that allows for more accurate estimates globally, by region, and over time. Also, there is little data on health care quality and access to procedures that have the potential to improve these outcomes.

To effect improvements in maternal, newborn, and child health (MNCH) outcomes, we need accurate epidemiological data about rates of death and morbidity, as well as the social determinants of health and processes of care. These data are needed from each country (and ideally region) to contextualise strategies. This is particularly important as countries are in different stages of the obstetric transition, with different health care infrastructures and priority needs [6]. Obtaining these data is currently hampered by numerous practical barriers, including a lack of standardisation of the predictor and outcome variables collected and their definitions [7]. There is keen interest in open-source software platforms, [8] self-programming and database revision.

The PRECISE (PREgnancy Care Integrating translational Science, Everywhere) Network is funded to collect epidemiological data and biological samples from women and babies in western (The Gambia), eastern (Kenya), and southern (Mozambique) sub-Saharan African countries. The aim is to understand, in the African setting, the predictors of placental disorders (hypertension, fetal growth restriction, and stillbirth) and determinants of their prognosis. The project will ‘deep phenotype’ around 10,000 pregnant women (and their babies), approximately 1500 of whom we anticipate will experience a pregnancy complicated by one of the placental disorders of interest (as above). (We will also collect data on around 1800 non-pregnant women of reproductive age as a control group.)

We have designed the PRECISE database with the following needs of the global research community in mind: (i) the movement away from purpose-built project databases so as to contain costs (initial and maintenance) and facilitate data-sharing [9]; (ii) the need for strategic support for large-scale, high-quality, epidemiological research; (iii) the desire to capitalise on the ubiquitous use of mobile technology for data collection in facility and in community; and (iv) the desire for open-access resources. We have built on previous work in outcome standardisation (by the International Consortium for Health Outcomes Measurement, ICHOM); data harmonisation (by the Global Pregnancy Collaboration, CoLab); the Manhiça Health and Demographics Surveillance System (HDSS), and an open-source, web-based health management information system (HMIS) platform (by the Norwegian Institute of Public Health).

In this manuscript, we describe our data collection platform of core and supplementary modules, with the potential to be used for high-quality epidemiological research. It is a single electronic repository of information (i.e. eRegistry) that can be shared by health system administrators, clinicians, clinical investigators, and discovery scientists through links with a laboratory information system.

## Methods

Given the focus of the PRECISE Network on placental disorders, the starting point was the CoLab ‘s COLLECT database, designed for global studies in pre-eclampsia [10]. COLLECT’s minimal and comprehensive data sets were reviewed for their appropriateness for data collection in our study sites, in terms of fields, definitions, and comprehensiveness for the purposes of the deep-phenotyping to be undertaken in PRECISE [11]. Also, we reviewed the software platform and connectivity requirements as we knew internet access to be intermittent.

In anticipation of the need for additional content and to enhance future usability, globally-recommended indicators in health management information systems for MNCH were identified and included, as component parts of key definitions (such as gestational age and blood pressure [BP] values), rather than reportable indicators that may require interpretation or judgement (such as a diagnosis of gestational hypertension) [12]. Variables were supplemented by review of the existing standardised data collection forms in each study site, for antenatal care (ANC), delivery, and ward care (antenatal or postnatal).

**Fig. 1:**
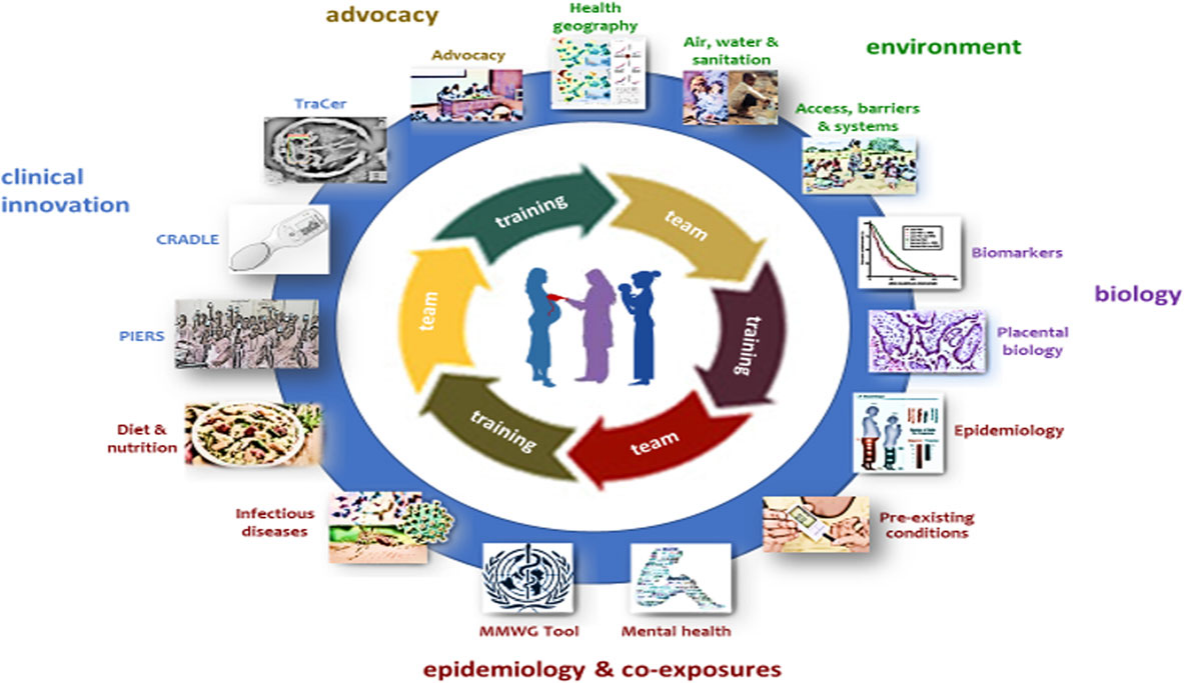
PRECISE holistic approach to pregnancy research

The content of supplementary modules was based on the holistic data needs of PRECISE (Fig. 1). These included the social determinants of health (e.g., socioeconomic status, air quality, geography, or nutrition); maternal pre-existing and infectious co-morbidities; maternal mental health; and elements of health systems’ strength (related to access, barriers, and quality of care). Standardised tools were identified whenever possible to address each of these data collection needs, based on needs-driven literature review (e.g., for maternal morbidity) and the experience of the PRECISE Network investigators.

The collection of biological specimens is recorded on an affiliated laboratory information management system (LIMS) described elsewhere. (For details of Baobab LIMS, please see Craik et al. Reprod Health 2019, in press [13].)

## Results

The PRECISE protocol specifies prospective data collection into the PRECISE database, as pregnant women are enrolled at facilities for: (i) routine ANC (i.e., ≈10,000 unselected pregnancies); or (ii) care of a placental disorder that is the focus of PRECISE (i.e., pregnancy hypertension, suspected fetal growth restriction [FGR], or intrauterine fetal death/stillbirth) if not already enrolled in PRECISE at that facility (i.e., ≈1500 women). (Additional control women of reproductive age will be enrolled when they present for contraceptive counselling or in the company of another person who is presenting for ANC or other care (i.e., ≈1800 women of reproductive age); in the Gambia, women of reproductive age will be recruited in the community.) [(For details of PRECISE Protocol, please see von Dadelszen et al. Reprod Health 2019, in press [14].) A project-specific eligibility screening has been designed to guide the study staff through the details of participant selection and data collection, documenting all information that would normally be recorded on a paper screening log, and eliminating the need for the study staff to carry paper guidance.

Depending on the site, not all staff have clinical training. However, all have been trained on the content and structure of the database, housed on android tablets (as described below). The sequence of data collection follows the flow of women through the clinic care system as much as possible.

### Data fields

In choosing the content of the PRECISE database, we reviewed all variables in the COLLECT ‘minimal’ or core data set and the ‘comprehensive’ version of the database. We supplemented our review with other MNCH instruments for measurement of demographics, pre-pregnancy health or obstetric history, processes of care, or outcomes.

The 923 variables in PRECISE were organised according into the following modules: (i) general visit information, including identifiers and geoinformatics variables of relevance; (ii) baseline demographics and social determinants of health; (iii) past medical and obstetric histories; (iv) nutrition, an additional social determinant of health; (v) current pregnancy details, including addiction and maternal mental health; (vi) details of assessments of pregnancy hypertension, FGR, and intrauterine fetal death (IUFD)/stillbirth; (vii) labour and delivery outcomes; (viii) maternal and newborn outcomes; and (ix) postpartum health and health care (Table 1). (Further details about the origins of each variable in each module are contained in Additional file 1, Tables S2A to S2N.)

**Table 1:** Content of modules

Modules	Description and tools
General visit information)	Identifiers for future contact^a^, including Health Demographic Surveillance System (HDSS) numbers, as relevant Geo-informatic variables of relevance, including from where the woman travelled to seek care, and how that travel was achieved (as performed in the Community-Level Interventions in Pre-eclampsia [CLIP] Mozambique Trial) [15, 16]
Baseline information & social determinants of health	Maternal demographics and pre-existing morbidities (Demographic Health Survey 7 [17], COLLECT database [11], and CLIP trials [18], informed by regionally-appropriate tribal categories in The Gambia, Kenya, and Mozambique Grameen Poverty Probability Index for Senegal (as a surrogate for The Gambia for which there is none), Kenya, and Mozambique [19] UN International Fund for Agricultural Development Multidimensional Poverty Assessment Tool, MPAT [20] Education and occupation (as in the CLIP trials [18]) Social supports and transport (as in the CLIP trials [18]) Joint Monitoring Programme on Water Supply and Sanitation (JMP), WHO and UNICEF Multiple Indicator Cluster Surveys (MICS)- Household Questionnaire 2017 [21]
Past medical and obstetric histories	COLLECT [11] CLIP trials [18] Demographic Health Survey 7 [17]
Nutrition	Dietary Diversity Score from the Healthy Life Trajectories Initiative (HeLTI) [22]
Current pregnancy details	COLLECT [11] CLIP trials [18] WHO recommendations on antenatal care for a positive pregnancy experience, 2016 [23] Maternal Death Notification Form – South Africa 2014 [24] Maternal mental health (WHO Maternal Woice Tool – antenatal care [25])
Details of placental disorders	COLLECT [11] CLIP trials [18] fullPIERS [26] and miniPIERS [27, 28] predictive models of adverse maternal and perinatal outcome in pre-eclampsia Details of stillbirth in INDEPTH standardised verbal autopsy tool [29]
Labour and delivery	COLLECT [11] CLIP trials [18] Adverse maternal outcome by Delphi consensus (fullPIERS [26] and iHOPE [30]) World Health Organization. WHO Recommendations on Antenatal Care for a Positive Pregnancy Experience. Geneva, Switzerland: World Health Organization, 2016 [23] Preterm birth phenotype [31] Intergrowth-21 standards for weight at birth [32] Averting Maternal Death and Disability (AMDD) Needs Assessment Toolkit: Modules 9 (Chart review for women with obstetric complications) and 10 (Chart review of newborn mortality) [33] ICD-MM [International Classification of Disease-Maternal Mortality (ICD-MM [34]) Maternal Death Notification Form – South Africa 2014 [24], Kenya 2017 [35] WHO 2011 Maternal Near-Miss Morbidity Approach [4] informed by an African Delphi Consensus process [36] WHO verbal autopsy tool for stillbirths [37], INDEPTH Standardized Verbal Autopsy questionnaire [29] International Classification of Disease-Perinatal Mortality (ICD-PM [38] Ministry of Health Perinatal Death Review Form – Kenya (2017) WHO Making Every Baby Count initiative [39]
Maternal and newborn outcomes	
Postpartum health	Post-traumatic Stress Disorder Checklist-Civilian Version, PCL-C [40] WHO Maternal Woice tool – postnatal care [41] (for mental health, violence against women and other maternal morbidity) WHODAS tool 2.0 [42] (health functioning) EN-SMILING tool [43] (early childhood development and infant nutrition) DHS-7 (also infant nutrition) [17]

^a^ To be stripped by encryption when data are transmitted to the central servers at UBC

The modules have the flexibility to be be grouped according to the data collection processes for PRECISE: screening, PRECISE visit 1, PRECISE visit 2, information from other ANC visits, pregnancy outcomes (birth, maternal, and newborn), and details of presentation with placental disease (as relevant) or with laboratory results (as applicable at any visit), as shown in Table 2.

**Table 2:** Presentation of data collection in PRECISE database

Icons in PRECISE (DHIS2)^a^	Timing of data collection	Applicable modules
	Screening (eligi-bility & data collection (each visit)	(Study or setting-specific)
	PRECISE visit 1	• General visit information • Baseline demographics and social determinants of health • Past medical and obstetric histories • Nutrition • Current pregnancy details
	PRECISE visit 2	• General visit information • Current pregnancy details
	Other ANC visit (each)	• Current pregnancy details (basic)
	Birth (labour and delivery, maternal and baby outcomes)	• Labour and delivery • Maternal and newborn outcomes
	Presentation with placental disease (any visit)	• Details of placental disorders
	Laboratory results (any visit)	(Relevant to all modules at any visit)

^a^ The REDCap interface has a ‘select event’ list

The 25 core components are focussed on intrapartum and immediate postpartum care as the time when most adverse outcomes cluster. The components would be appropriate for future use as screening data collection and would impose the lowest burden on the health system. These cover globally-recommended indicators in national HMIS recording and reporting forms, as per a review of data elements related to maternal and newborn health that were captured at different levels of the health system by HMIS tools from 14 USAID priority countries [12]. These core components consist of: (i) key mortality outcomes, of maternal death, stillbirth, and neonatal death (early and late); and (ii) life-saving interventions, of Human Immunodeficiency Virus (HIV) testing, BP measurement, iron therapy, uterotonic use after delivery, postpartum maternal assessment within 48 h of birth, and newborn resuscitation, immediate skin-to-skin contact, and immediate drying. Additional variables complete the 17 core administrative variables for the mother and baby. For the mother, these are: maternal age, parity, weight, substance use, need for intensive care as reflected by maternal near-miss morbidity, transfusion of blood products, and maternal length of stay. For the baby (ies), the core administrative variables are: spontaneous or iatrogenic preterm birth, sex, birthweight, birth injury, and obvious major birth defects and broad type), as well as location and mode of delivery [44]. These outcomes were developed by the ICHOM. To date, we have not included patient-reported health status, satisfaction with care, health care responsiveness, and birth experience, all of which would require specific data collection tools and be hampered by low health literacy at our study sites.

Given the deep-phenotyping mandate of PRECISE, these core components were supplemented by additional variables suitable for the immediate purpose of deep-phenotyping in PRECISE, as well as for future use in different locations (depending on needs and interest) or at different times (to monitor trends, depending on regional/national/global needs). The variables were defined according to various context-specific sources. COLLECT definitions were used whenever possible, for the 60/923 variables shared with the COLLECT minimal dataset; however, definitions were not always deemed feasible to use from COLLECT given response options that were not appropriate for our study settings (e.g., ethnicity categories that could not be mapped to context-specific options), unavailability of the intervention (e.g., invasive prenatal diagnosis), and/or the absence of robust clinical records (e.g., family history of pre-eclampsia). A particular concern was the use in COLLECT of some over-arching stem questions (such as occurrence of “maternal morbidity”) that relied on high health literacy of the data collectors who only if answering ‘yes’, would see questions about specific morbidities; health literacy is variable among women, care-providers, and data collectors in our study settings. In contrast, we focussed on clinical data that care-providers routinely document, not “reportable indicators”; for example, care-providers record the BP measurement and use the system-generated date and gestational age to autogenerate a diagnosis of gestational hypertension at 20 weeks. This general principle was respected throughout, such as for maternal and neonatal near-miss morbidity [4, 5, 45].

To ensure high-quality of data, programme rules were added to implement skip-logics and cross-validation rules for checking inconsistencies in real time. Each module has a ‘See details’ option, the programming of which is customised to provide the key details of relevance to the user.

The woman’s study identifier is used to link her epidemiological data with biorepository samples tracked in Baobab LIMS, the LIMS chosen for the project given it is open-source and its development to facilitate harmonisation of biobanks across Africa, our geographical focus. In this way, a participant’s personal health information is not attached to any specimen in the Baobab LIMS database. Executable, customised programmes have been created to be run periodically by the data manager to transmit biobanking core data into the database. (For details of Baobab LIMS, please see Craik et al. Reprod Health 2019, in press [13].)

### Software

The PRECISE database is based on the District Health Information System 2 (DHIS2) software and its application, *Tracker*. First, DHIS2 is widely used (by 67 low and middle-income countries) and, importantly, has been implemented in the Palestinian Territories as the national eRegistry for maternal and child health [46]. The *Tracker* application provides capacity for individual-level data entry. Second, *Tracker* is flexible, easily set-up, open-source, and accessed using a variety of tools, including android tablets and smartphones, to maximise flexibility of data collection at service delivery points [47]. If internet connectivity is lost, work can continue offline to facilitate efficient and effective data management. There is a ‘share’ function that generates a QR code (i.e., a machine-readable optical label that contains the relevant information about a PRECISE participant) that, if scanned by another device, will transfer the woman’s information to another PRECISE tablet, allowing for data collection without interruption. Finally, the mandate of PRECISE includes capacity-building in Africa, and DHIS2 *Tracker* has the greatest potential to form the basis for an eRegistry. DHIS2 is packaged as a standard Java Web Archive file and runs on any Servlet container with Java Runtime Environment version 8 or higher installed, including the need for a web server and a database server with sufficient memory and storage space. As the preferred software environment for production server, Ubuntu 16.04 LTS operating system, PostgreSQL database and Tomcat Servlet are recommended. Training materials have been written so that those entering data have requisite skills to ensure accuracy [47].

Other software was considered. We found the structure of the COLLECT database challenging for the purposes of PRECISE. First, we had available to us either all of the field labels (variables and their characteristics) in the minimal dataset (i.e., ≈500) or all in the comprehensive dataset (i.e., ≈5000), but neither approach was realistic due to challenges with individual variables, as outlined above. Second, the MedSciNet software required continuous connectivity for data entry, but this is not available at our sites. We considered *Open Data Kit*, a mobile data management application that has been widely deployed in more than 130 countries [48]; however, the application (app) cannot handle frequent changes to the data model and we sought a resilient system for future use [49]. We considered *Open Medical Record System*, created as an open-source health software for low-resource settings [50]; while widely-used for HIV care, this is not the case for antenatal and postnatal care of mothers and babies. Finally, we considered Research Electronic Capture (REDCap) software, as it is widely used and open-access; however, REDCap does not have a personal dashboard, may not support well repeatable instruments and events, and the website has only a report tool with filters, rather than analytical tools needed for future functionality.

**Fig. 2:**
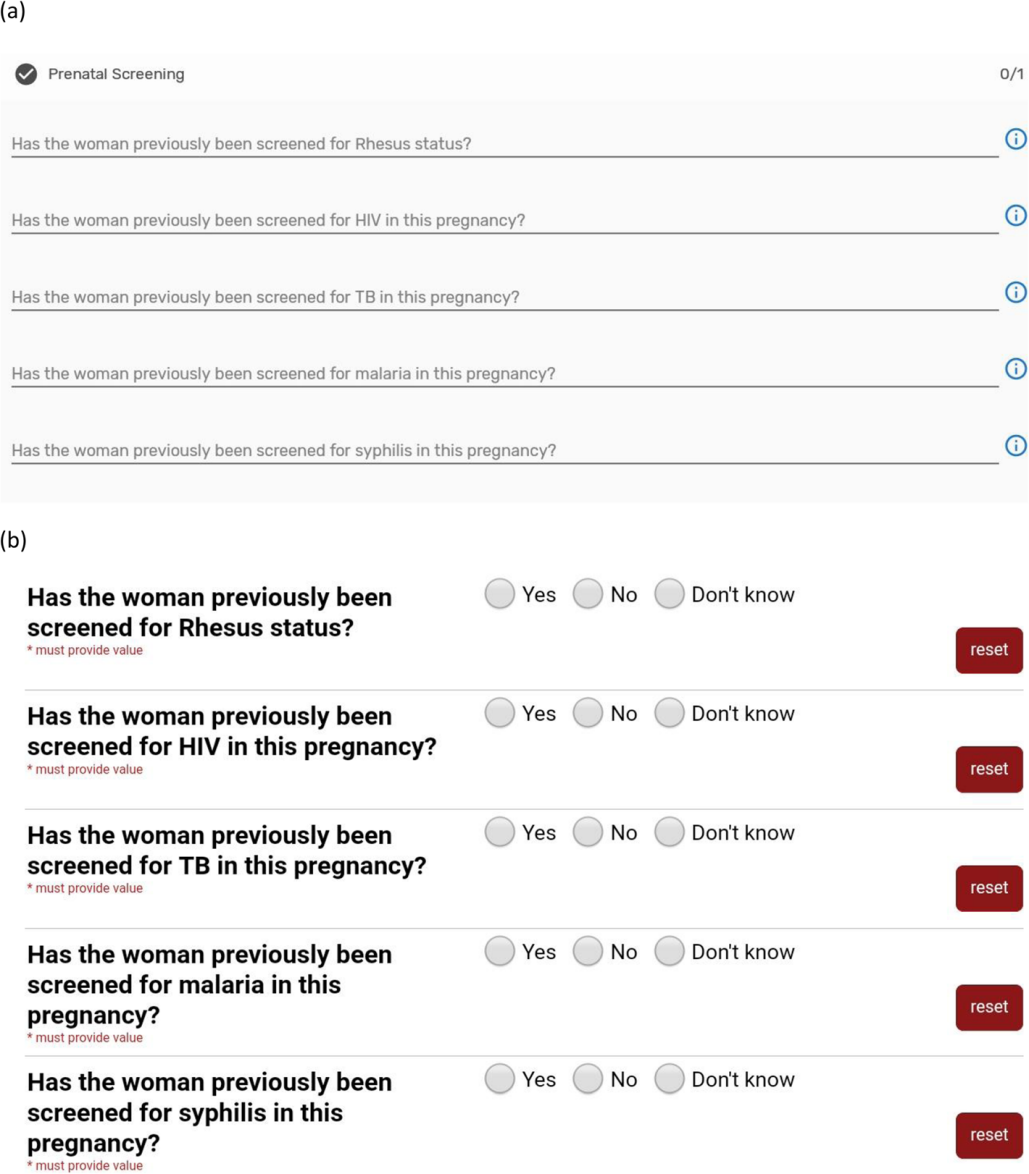
Interfaces for (a) DHIS2 Tracker and (b) REDCap

We did experience difficulties with the very large volume of questions to be asked in PRECISE which caused the DHIS2 Android Capture app to perform very slowly; a work-around was created to decrease the volume of data collection associated with any one ‘icon’ (as in Table 2), and this dramatically improved speed. However, in testing, the software was still not syncing 100% of all data entered, a problem experienced by other software users. In addition, the personal dashboard of a participant across multiple programmes (or tiles) was not always updated automatically if it was not opened in a programme. Given our need to start PRECISE data collection, we opted to begin on REDCap software that is compatible with the technical specifications of DHIS2 with which it has a similar look and feel (Fig. 2). Further testing will determine whether we revert to direct use of DHIS2, or remain with the REDCap interface.

In the PRECISE database, all participants are given a unique study identifier. Personal information is collected and held on a local database kept in a locked environment, with direct access restricted to authorised individuals who are responsible for monitoring and backing up the system. All other access to the database is controlled by logical security with access control and across a standard secure Hyper Text Transfer Protocol Secure network protected by a firewall and system controls with authentication, role-based authorisation, and audit trails. Strong password policies include password length and complexity restrictions. All database back-ups are encrypted and local Information Technology security policies and procedures followed.

Data collection tablets are password-protected and back-ups are encrypted. Each user logs into the app with a strong username and password, ensuring the study participant’s confidentiality even with loss of tablets. Data are synchronised with local servers via secure connections, preferably daily. To ensure confidentiality, only authorised staff have access to personal information to facilitate data collection and cleaning. Loss or damage of a tablet that results in loss of data would be reported immediately. Information would be erased before any damaged tablets were recycled. Only password-protected computers running an antivirus program are used to download and process personal information for data linkage and cleaning.

All data are stored locally on high capacity servers stored in locked rooms. Extra measures for security include auxiliary power and continuous system monitoring. A tape back-up system is used for the database (and website).

A copy of all de-identified qualitative, clinical, geo-informatic, and laboratory data are stored on a remote server located at the British Columbia Children’s Hospital Research Institute with daily backup and management by the PRE-EMPT team (PREgnancy Evidence, Monitoring, Partnerships and Treatment) at the University of British Columbia (UBC). Data are stored on a secure database on a high-speed network with access control, and servers are stored in a secure locked server room with restricted access to authorised system administrators. When data are sent for merging with master databases, research staff will keep a copy on their restricted network drive. The final dataset along with hard copies of results will be kept for at least 10 years.

For PRECISE, in each country, the database system is hosted and managed by local IT system administrators who are responsible for network security and access control, system updates, database back-up and recovery, and the in-country computer updates, in compliance with local IT network, system, and security policies. The data manager has set up and configured the database, tested the system functionalities, updated ‘meta-data’ (that help users find relevant information within the database), and installed/configured mobile apps on the tablets. The data manager is responsible for importing and exporting data, monitoring data updates, audit trails and reports, and resolving data queries within the study team.

It is the duty of sites to ensure data collected are complete and accurate and to run validation rules. Data are queried periodically by the local data manager to check for timely data collection and synchronisation, missing information, and discrepancies. Only de-identified data will be transmitted to the UBC central database, preferably on a weekly basis, through a secure connection with 256-bit Secure Sockets Layer encryption. At UBC, data queries are run and a query report with feedback provided to each site’s local data managers. The data query report focusses on missing values without reasonable comments, out-of-range values, and data inconsistencies that violate validation rules. The report is designed to be reviewed and the queries resolved with the local team. Built-in audit trails provide historical records of data-update activities.

Compliance with General Data Protection Regulation (GDPR) is reliant on both the database and the operational policies, procedures and processes of the local study teams, to ensure that the necessary steps are taken to: obtain appropriate informed consent for data management (including collection, retention, sharing, disposal, and dispute); staff training; designation of a Data Protection Officer; tablet protection; server/platform protection; and detection and reporting of breaches. In addition, consent to participate in any project involving the database must protect participants’ eight individual rights under GDPR to: be informed, access, rectification, withdrawal of consent, erasure, restriction of processing (of data), data portability, and objection.

### Challenges

Our challenge related to our planned software platform, DHIS2, has been discussed above. Additional challenges relate to implementation of database content.

First, the World Health Organization (WHO) ANC guidelines recommend that, “Health-care providers should ask all pregnant women about their tobacco use (past and present) and exposure to second-hand smoke as early as possible in the pregnancy and at every antenatal care visit”, and, “Health-care providers should ask all pregnant women about their use of alcohol and other substances (past and present) as early as possible in the pregnancy and at every antenatal care visit” [23]. Our in-country teams customised these questions to deal with cultural sensitivities.

Second, given the critical importance of mental health in pregnant and postpartum women, we had planned to integrate the WHO ‘WOICE’ tool for measurement of maternal morbidity, to which we added a scale of post-traumatic stress disorder [25, 41]. However, in-country teams were not comfortable due to the lack of both privacy in which to ask and answer these questions in the ANC setting, as well as the lack of well-established care pathways for women identified as having issues or being at high risk; antenatal and postnatal modules containing these questions are currently offline while we undertake care pathway mapping to prepare for the inclusion of these questions during the course of the project.

## Discussion

The PRECISE database as designed is a unique infrastructure of a generic, unified data collection platform of core and supplementary variables that cover globally-recommended indicators included in HMIS recording and reporting forms, comprised of: key outcomes, life-saving interventions, and an additional 17 core administrative variables for the mother and babies. In addition, the database has a suite of additional variables designed for ‘deep phenotyping’ related to the placental disorders of hypertension, FGR, and stillbirth, but which are widely applicable for studying pregnancy-related conditions: social determinants of health, maternal co-morbidities, nutrition, mental health and domestic violence. The data elements and definitions have been standardised to comply with existing data dictionaries and/or tools, from international clinical, research, and policy organisations, including the WHO, as well as efforts for international outcomes standardisation. The software platform is DHIS2, which is open-access and supported by free programming training by the University of Oslo as a WHO Collaborating Centre.

### Strengths and limitations

Data collection at the point of clinical care is enabled by the modular structure of the PRECISE database. This approach to an electronic health record is both longitudinal and truly prospective, so collection of relevant clinical data will precede outcomes, which means that they cannot be biased by those outcomes. Specifically, predictors will be collected prior to occurrence of a placental disorder, and a placental disorder prior to the occurrence of maternal and perinatal mortality and morbidity. This is critical to understanding the epidemiology and true denominator and numerator of adverse outcomes, such as stillbirths, a major contributor to lives lost in Reproductive MNCH (RMNCH).

DHIS2, on which *Tracker* is programmed, is sustainable. The core software development team is hosted as a “global public good” at the University of Oslo, a world leader in HMIS strengthening in less-developed countries; DHIS2 has been implemented, at least regionally, in 87 countries and Indian states, with 53 operating at national scale. The University of Oslo also contributes by offering in-country capacity building and implementation support and research, and as a WHO Collaborating Centre for Innovation and Implementation Research.

Limitations of the PRECISE database include how the questions must be structured; any responses for which ‘mark all that apply’ is appropriate must be phrased as different questions. Fields cannot currently be auto-filled from earlier data entries on the Android app; for example, if a woman has indicated that she is HIV positive on a previous form, Tracker will not automatically fill in later fields recording her HIV status. Also, images from supporting documentation (such as visual aids to describe sanitation facilities) cannot currently be uploaded and stored on the DHIS2 Android app. There is no offline data capture app that is supported on an Apple operating system device; however, the platform can still be accessible via internet through Safari or Chrome browsers if installed on an Apple device.

### The literature

The WHO promotes client registry-based health information systems as one of the most promising avenues to support quality universal health coverage in RMNCH. While most less-developed countries have national eHealth plans, a lack of harmonisation and capacity-building risks squandering opportunities for research and collaboration.

A standardised approach to RMNCH data field collection is critical. Measurement strategies must be consistent across communities and countries, and over time, in order to benchmark and address underlying differences, improve outcomes and reduce disparity. The Bill & Melinda Gates Foundation (BMGF)-funded *Healthy Birth, Growth, and Development – knowledge integration* initiative is assembling ‘big data’ and novel analytic data rallies (focussed analytic efforts) from more than 170 BMGF-funded projects, involving ≈12 million subjects from 34 countries, to inform key questions in child health and development [51]; the investigators will drill down through masses of data looking for patterns, statistical anomalies, and other newly discovered facts to inform new hypotheses in clinical research, and fashion updated clinical guidelines and protocols [52]. However, the initiative faces the challenge of different data fields, with different definitions, and different platforms – the same challenge faced by individual patient data meta-analyses in pregnancy. For example, in its synthesis of international angiogenic biomarker data (22 pregnancy cohorts, 16,462 pregnancies), the BMGF-funded CoLab estimates that more than 300 h were required to map data fields and definitions from one cohort to another, in order to find common ground on which to base the data synthesis [53].

Electronic health (eHealth) technology is the most frequently cited opportunity for maternal health among international researchers [54]. eHealth has been described as the … use of information and communications technologies in support of health and health-related fields, including health care services, health surveillance, health literature, and health education, knowledge, and research [52]. At a global level, health data may: (i) not be captured at all; or if collected, (ii) captured but multiple times in multiple ways, or (iii) captured but in a way that cannot be shared due to interoperability issues or a lack of standardised definitions. This is not surprising given that approximately half of all referral facilities rely on paper data management [30]. The implications are illustrated by estimates of the maternal mortality ratio (per 100,000 live births) in Lesotho that vary by over 10-fold depending on the data source– 100/100,000 based on their confidential enquiry into maternal deaths, 500/100,000 according to entry of aggregate data from a paper birth registry into DHIS2, and 1057/100,000 as reported by the Demographic Health (household) Survey [Personal communication, B Pattinson 2018 Oct 4].

Making data valuable to the collector is the key to obtaining quality data, and lack of data use where it is collected has been cited as the weakest part of the HMIS in many countries [55]. In contrast, collecting data at point-of-care, in an electronic record with decision- and work-flow support, is designed with care providers in mind as both users and beneficiaries.

While many countries are implementing health registries in various forms, very few can act as an integrating backbone for health information and bioscience, clinical, and epidemiological research [56]. This is unrelated to the fact that the majority are in transition from paper-based data to digital data collection systems, but rather that even when fully implemented, the current bespoke ‘top-down’ solutions may not capture locally-relevant determinants of outcomes or be inter-operable between countries. Additionally, local/regional ‘bottom-up’ efforts or bespoke databases for specific research projects are not standardised and thus, unable to monitor national trends [52].

eRegistries are systems using information and communication technologies for the *systematic* longitudinal collection, storage, retrieval, analysis, and dissemination of uniform information on the health determinants and outcomes of individual persons, to serve diverse stakeholders, including those providing health care services or health surveillance, and those conducting research [56]. eRegistries are feasible and have been successfully implemented in MNCH [56]. Most undertake duplicate data collection on paper with transfer to an electronic database (e.g., Maternal Newborn Health Registry of the National Institutes of Health and BMGF-funded Global Health Network). However, some countries (e.g., Kenya) have published research based on primary data entry in fully electronic registries at national or regional levels, and individual records have also been linked with laboratory tests and biorepositories [56, 57], and a fully-functional eRegistry programmed on DHIS2 with decision support is currently being evaluated in the Palestinian Territories [46].

The strengths and operational challenges of DHIS2 have been recently reviewed systematically, in 20 studies from 11 countries [47]. The most important strengths were the technical features of the software (e.g., open-source); proper management of data (e.g., data entry at point of service provision); and application flexibility (e.g., ability to customise to meet local needs and sustainability of software); all are being leveraged in PRECISE. The most important operational challenges were management/leadership (e.g., proper planning for establishment) and training (e.g., of staff), sufficient workforce, appropriate communication infrastructure (e.g., internet), and the political, cultural, social and structural infrastructure (including linguistic challenges). All of these are being addressed in PRECISE, by staff hiring, training sessions and materials, close supervision, provision of enhanced internet capabilities (although not continuous), and a strongly supportive leadership, including local governmental officials.

While follow-up in PRECISE is currently to 6 weeks postpartum, it is critical to develop mechanisms to facilitate long-term follow-up. Conducting PRECISE in collaboration with the Manhiça Health Research Centre affords us the opportunity to link our database with the routine morbidity surveillance conducted for all children under 15 years of age who attend for inpatient or outpatient care in the area covered by their HDSS [58].

Finally, there is a need to understand which component(s) of packages of care are key elements of successful interventions. For example, the WHO eight-contact ANC model includes health promotion and nutritional interventions, and prevention and early detection of selected pregnancy-related conditions and concurrent diseases, including malaria, HIV, and tuberculosis. There are currently significant knowledge gaps that impede optimisation of care, given: the complexity of factors to consider; the need for countries to adapt recommendations based on their country context and populations’ needs; and the inability of rigorous study methods (such as randomised trials) to evaluate each individual component of such packages, particularly in various settings. However, the volume and quality of data collected in an eRegistry can facilitate an understanding of the effects of multiple mediators, while accounting for unobserved confounders, using a counter-factual approach with multiple simulations from observed data, and bootstrapping for confidence intervals. Taking the eight-visit WHO ANC model, one could ask if it is necessary to measure proteinuria at every ANC visit? Or, what is the value of screening for hypertension at 32 weeks’ gestation, in relation to other potential mediators of any observed effect (Fig. 2)?

## Conclusions

We seek to build on PRECISE (in Africa) and the eRegistry concept by developing a generic, unified data collection platform that links clinical research and clinical care, thereby creating both a ‘learning health system’ for care-providers, and a monitoring, learning and evaluation system for academics and health administrators. Research could take the form of large-scale epidemiological work, clinical trials, implementation science, or discovery science by linking clinical data with stored biological samples logged in a LIMS system. Distributed analytical software packages (such as DataSHIELD) can now enable real-time, in-country analyses and research collaborations. In addition, there is potential to integrate decision support, facilitate remote access and analyses, and replace manual aggregation of data from multiple sources with electronically-generated reports for submission to central authorities, thus providing a mechanism for governments and other international agencies to monitor trends and identify priorities for more detailed data collection.

## Supplementary information

**Additional file 1.** Supplementary Database Tables S2A-S2N.

## Data Availability

The PRECISE Network Study are owned by the in-country teams. The PRECISE Network Data and Sample Access Committee, on which each in-country Principal Investigators (or designate) shall serve, shall provide guidance and oversight on the collection, processing and utilisation of specimens and data collected on behalf of the PRECISE Network Study.

## Abbreviations

ANC: Antenatal care
App: Application
BMGF: The Bill & Melinda Gates Foundation
BP: Blood pressure
CoLab: Global Pregnancy Collaboration
DHIS2: District Health Information System 2
eHealth: Electronic health
eRegistry: Electronic registry
FGR: Fetal growth restriction
GDPR: General Data Protection Regulation
HDSS: Health and Demographics Surveillance System
HIV: Human Immunodeficiency Virus
HMIS: Health management information system
ICHOM: International Consortium for Health Outcomes Measurement
IUFD: Intrauterine fetal death
LIMS: Laboratory information management system
MNCH: Maternal, newborn, and child health
PRECISE: PREgnancy Care Integrating translational Science Everywhere, Network
PRE-EMPT: PREgnancy Evidence, Monitoring, Partnerships and Treatment
REDCap: Research Electronic Capture
RMNCH: Reproductive MNCH
UBC: University of British Columbia
WHO: World Health Organization
