# Supplementary material for: The PRECISE (PREgnancy Care Integrating translational Science, Everywhere) database: open-access data collection in maternal and newborn health
Source: Reprod Health. 2020 Apr 30;17(Suppl 1):50. doi: 10.1186/s12978-020-0873-8 (PMC7191679; doi:10.1186/s12978-020-0873-8)
Supplement: Supplementary file 1 — Additional file 1. Supplementary Database Tables S2A-S2N. [file 12978_2020_873_MOESM1_ESM.docx]

**SUPPLEMENTARY DATABASE TABLES**

**Table S2A: General Visit Information**

**Table S2B: Baseline/1^st^ PRECISE visit information**

**Table S2C: Past history**

**Table S2D: Dietary Diversity Score screen**

**Table S2E: Current pregnancy details**

**Table S2F: Additional questions for MMWG to be asked only at the 2^nd^ PRECISE visit, in the third trimester**

**Table S2G: Time of Disease (ToD) pregnancy**

**Table S2H: Delivery suite module**

**Table S2I: Maternal outcomes and care**

**Table S2J: BABY information (for each stillborn and liveborn)**

**Table S2K: Woman’s Discharge (all live women, to be completed at the time they leave the facility)**

**Table S2L: Infant(s) Discharge (all live babies, to be completed at the time they leave the facility)**

**Table S2M: Laboratory**

**Table S2N: Withdrawal/Lost to follow-up**

**Abbreviations**

AC (abdominal circumference)

AFI (amniotic fluid index)

AKI (acute kidney injury)

ALT (alanine aminotransferase)

ANC (antenatal care)

ART (antiretroviral therapy)

AST (aspartate transaminase)

BMI (Body Mass Index)

BP (blood pressure)

BPM (beats per minute)

BPD (biparietal diameter)

BW (birthweight)

C-section or CS (Caesarean)

CNS (central nervous system)

COD (cause of death)

Comm (community)

CPAP (continuous positive airway pressure)

CPD (cephalopelvic disproportion)

CRL (crown-rump length)

dBP (diastolic blood pressure)

DM (diabetes mellitus)

DK (don’t know)

ECG (electrocardiogram)

EDD (expected date of delivery)

EDTA (Ethylenediaminetetraacetic Acid)

EDV (end diastolic velocity)

ETT (endotracheal tube)

FGM (female genital mutilation)

FGR (fetal growth restriction)

FHR (fetal heart rate)

FL (femur length)

GA (gestational age)

GBS (Group B Streptococcus)

GDM (gestational diabetes mellitus)

GH (gestational hypertension)

GI (gastrointestinal)

GU (genitourinary)

HC (head circumference)

HDP (Hypertensive Disease of Pregnancy)

HIV PCR (HIV Polymerase Chain Reaction test)

HPV (Human Papillomavirus)

ICD-MM (International Classification of Disease-Maternal Mortality)

ICU (intensive care unit)

INR (international normalized ratio)

IUFD (intrauterine fetal death)

LLITN (long-lasting insecticide-treated net)

LMP (last menstrual period)

MAP (mean arterial pressure)

MDNF SA (Maternal Death Notification Form – South Africa)

MDR (Maternal Death Review)

MgSO4 (magnesium sulphate)

MMWG (Maternal Morbidity Working Group tool)

N (number)

NND (neonatal death)

PDR (Perinatal Death Review)

PET (pre-eclampsia)

PHC (primary health centre)

PI (pulsatility index)

PPH (postpartum haemorrhage)

PTSD (post-traumatic stress disorder)

RBC (red blood cells)

RDT (rapid diagnostic test)

RI (resistance index)

RR (respiratory rate)

SA (spontaneous abortion)

SB (stillbirth)

sBP (systolic blood pressure)

SES (Social and Economic Status)

SFH (symphysis-fundal height)

SGA (small for gestational age)

STI (sexually transmitted infection)

T (temperature)

TA (therapeutic abortion)

TB (tuberculosis)

TBV (total blood volume)

TCD (transcerebellar diameter)

ToD (time-of-disease)

UBC (University of British Columbia)

UBT (uterine balloon tampenade)

Ut RI (uterine artery resistance index)

Ut PI (uterine artery pulsatility index)

VTE (venous thromboembolism)

WCC (white cell count)

WRA (women of reproductive age)

**Table S2A: General Visit Information**

| **IDENTIFIERS** |
| --- |
| **Interviewer/data collector ID** *(text box)* |
| **What is your name?** *(text boxes for forename and family name)* (will be stripped when sent to UBC) |
| **What is your national ID number/HDSS number?** *(text box)* |
| **What is your PRECISE Study ID number *(number box)**** (Text ID to woman using DHIS2 text function?) – will autopopulate after 1^st^ visit |
| **What is the woman’s PRECISE Study ID number *(number box)* Cohort 5 only** |
| **What is the name of the health facility where the woman is being seen?*** *(Kenya – Rabai, Mariakani; Mozambique – Xinavane, Manhica; The Gambia – Farafenni, Illiassa, Ngenyen Sanjal,) -* will autopopulate after 1^st^ visit |
| **Is there a mobile phone that we could reach you on?** *(yes - own phone, yes - husband’s phone, yes - other person’s phone, no)* (will be stripped when sent to UBC) |
| 🡪 If yes, what is the number? NB. Preference for ‘yes – own phone’ *(Number box)* |
| **Is there another mobile phone that we could reach you on?** *(yes - own phone, yes - husband’s phone, yes - other person’s phone, no)* (will be stripped when sent to UBC) |
| 🡪 If yes, what is the number? *(Number box)* |
| **Which village do you live in?** *(drop-down options which are appropriate to each site)* |
| **What is the address of where you currently live?*** *(text box)* |
| **GPS Coordinates of woman’s home** |
| **Where did you come from when you arrived at this facility**?* *(Home, work, friend’s home, health facility, other (specify))* |
| 🡪 If health facility, record name of health facility (*text box)* |
| **How did you get here today?*** *(Drop-down that asks to tick ALL that apply: on foot, own bicycle, own motorbike, own car, public bus, lift in friend-relative’s car, lift on friend-relative’s motorbike, lift on friend-relative’s bike, taxi-car, taxi-motorbike, tuk-tuk - auto-rickshaw, other (please specify))* |
| **How long did it take you to get here?*** *(<15 mins, 15-30 mins, 30 mins- 1hr, >1hr)* **Cohorts 1, 2, 3 and 4 only** |

**Table S2B: Baseline/1^st^ PRECISE visit information**

| **Demography** |
| --- |
| **In what year were you born?** *(yyyy, don’t know)* |
| 🡪 if *don’t know* for year, how old were you at your last birthday? *(text box for age in years).* |
| **In what month were you born?** *(month, don’t know)* |
| 🡪 *don’t know* month? *(yes, no)* |
| **What is your ethnicity?** *[****Caucasian*** *(Origins in Europe, Middle East, North Africa [Arabic origins], Western Russia [including Afghanistan and South Russia] and Hispanics of European origin),* ***Black African*** *(**Origins in any of the original peoples of Africa), in* *The Gambia Mandinka, Wollof, Sarahule, Jola, Fula, Other – specify, in Kenya Kalenjin, Kamba, Kikuyu, Kisii, Luhya, Luo, Maasai, Meru, Mijikenda/Swahili, Somali, Taita/Taveta, Turkana, Samburu, Other – specify, and in Moz* *Makonde, Yao, Makhuwa, Shona, Tsonga (Ronga, Changane, Tswa), Chope, Bitonga, Chuwabo, Sena, Nyungwe, Other – specify),* ***Asian*** *(Origins in the Indian sub-continent [e.g., India, Pakistan, Bangladesh and Sri Lanka], or in the Far East and Southeast Asia [e.g., China, Japan, Korea, Philippines, Thailand, Eastern Russia]),* ***Other*** *(textbox,* *includes origins not represented above, such as Inuit, Maori, Australian Aborigine, North and South American Native Peoples, Hispanics of Caribbean, Central & South American origin, and Pacific Islanders)]* |
| **What is your religion?** *(Muslim, Christian, Traditional/spiritualist/animist, Hinduism, Sikhism, Buddhism, Judaism, Parsi/Zoroastrian, Donyi-Polo, Sanamahi, Baha’i, Bisaka group/Faith of Unity, Kirat, Other (specify), None)* |
| **Education** |
| **What is the highest level of school you attended?** *(none, primary, secondary, higher)* |
| **Work/study** |
| **What kind of work do you do?** (*housewife, student, professional, factory, large-scale agriculture, market trader, construction, other(specify))*  If more than 1, select the main one) |
| **Living Situation and male partner** |
| **Do you currently live alone?** *(yes, no)* |
| 🡪 if no, do you currently live with a partner? *(**yes, no)* |
| 🡪 if no, do you currently live with your own parents? *(yes, no)* |
| 🡪 if no, do you currently live with parents-in-law? *(yes, no)* |
| 🡪 if no, do you currently live with other relatives? *(yes, no)* |
| 🡪 if no, do you currently live with friends? *(yes, no)* |
| 🡪 if no, do you currently live with your own children? *(yes, no)* |
| 🡪 if no, do you currently live with other children? *(yes, no)* |
| 🡪 if no, do you currently live with people other than the ones listed? *(yes, no)* |
| 🡪 If yes, please specify *(text box)* |
| **How many members does the household have?** *(1, 2, 3, 4, 5, 6, 7, 8, 9 or more)* |
| **What is your current marital status**? *(Never married (or single), co-habiting, currently married (and only wife), currently married (one of two or more wives), separated, divorced, widowed)* |
| 🡪 If currently married or co-habiting, how long have you and your husband/partner been together? *(yyyy/mm), if less than a year must enter 00 in years box)* |
| **Home environment** |
| **What is the primary construction material of the housing unit’s exterior walls?** *(Stone & mortar/ metal sheeting/reinforced concrete/brick/cement bricks/logs/earth/mud or earth bricks/mud & straw/wood sheeting/tin, cardboard, paper or sacks/thick plastic/thin plastic/reeds, sticks, bamboo or palm/thick fabric/thin fabric/other (specify)* |
| **What is the primary fuel source your household uses for heat?** *(None/low-voltage electricity from grid (legal or illegal connection)/medium or high-voltage electricity from grid (legal or illegal connection)/electricity from generator/electricity from solar cells, wind turbine or small hydroelectric dam/liquid fuel (petrol, kerosene)/gas fuel (methane from tank, biogas)/coal or charcoal/vegetable or animal-based fats or oils/paraffin wax or battery-powered source/wood, sawdust, grass or other natural material/heat not needed in region/don’t know)* |
| **What is the primary fuel source your household uses for cooking?** *(None/low-voltage electricity from grid (legal or illegal connection)/medium or high-voltage electricity from grid (legal or illegal connection)/electricity from generator/electricity from solar cells, wind turbine or small hydroelectric dam/liquid fuel (petrol, kerosene)/gas fuel (methane from tank, biogas)/coal or charcoal/vegetable or animal-based fats or oils/paraffin wax or battery-powered source/wood, sawdust, grass or other natural material/don’t know)* |
| **What is the primary fuel source your household uses for lighting?** *(None/low-voltage electricity from grid (legal or illegal connection)/medium or high-voltage electricity from grid (legal or illegal connection)/electricity from generator/electricity from solar cells, wind turbine or small hydroelectric dam/liquid fuel (petrol, kerosene)/gas fuel (methane from tank, biogas)/coal or charcoal/vegetable or animal-based fats or oils/paraffin wax or battery-powered source/wood, sawdust, grass or other natural material/don’t know)* |
| **What is the main source of drinking water used by members of your household?** *(Piped into dwelling/piped into yard or plot/piped to neighbour/public tap or standpipe/tubewell or borehole/protected dug well/unprotected dug well/protected spring/unprotected spring/rainwater/tanker-truck/cart with small tank/water kiosk/ surface water (river, dam, lake, pond, stream, canal, irrigation channel)/bottled water/ sachet water/other (specify)* |
| **What is the main source of water used by members of your household for other purposes such as cooking and handwashing?** (If unclear, probe to identify the place from which members of this household most often collect water for other purposes) *(Piped into dwelling/piped into yard or plot/piped to neighbour/public tap or standpipe/tubewell or borehole/protected dug well/unprotected dug well/protected spring/unprotected spring/rainwater/tanker-truck/cart with small tank/water kiosk/ surface water (river, dam, lake, pond, stream, canal, irrigation channel)/bottled water/sachet water/other (specify)* |
| **Where is that water source located**? (*In own dwelling, in own yard or plot, elsewhere)* |
| **How long does it take for members of your household to go there, get water and come back?** (*Members do not collect, number of minutes, don’t know)* |
| 🡪 Duration in minutes *(number box)* |
| **What kind of toilet facility do members of your household usually use?** *(Flush or pour flush toilet, Ventilated improved pit latrine, Pit latrine with slab, Pit latrine without slab, open pit, Composting toilet, Bucket, Hanging toilet or hanging latrine, No facility or bush or field, Other (specify))* |
| 🡪 If ‘Flush’ or ‘Pour flush’, where does it flush to?  *(**Piped sewer system, Septic tank, Pit latrine, Open drain, don’t know where)* |
| **Where is this toilet** **facility located?** *(In own dwelling, in own yard or plot, elsewhere)* |
| **Do you share** **this facility with others who are not members of your household?** *(yes, no)* |
| **Where do you or other members of your household most often wash your hands?** *(Fixed facility (sink or tap) in dwelling, fixed facility (sink or tap) in yard or plot, Mobile object (bucket or jug kettle), No handwashing place in dwelling or yard or plot, Other (specify))* |
| **Do you have any soap or detergent or ash/mud/sand in your house for washing hands?** *(yes, no)* |
| **Additional Grameen PPI – ALL COUNTRIES** |
| **Does your household own any irons?** *(yes, no)* |
| 🡪 *if yes, type of iron (electric, non-electric, charcoal)* |
| **Additional Grameen PPI – KENYA & MOZAMBIQUE ONLY** |
| **What is the main material of the floor of your home (excluding kitchen and bathrooms)?** *(Uncovered or other, packed earth, wood or parquet, marble or granite, cement, mosaic or tile)* |
| **Additional Grameen PPI – KENYA ONLY** |
| **What is the highest school grade that the female head/spouse has completed?** *(None or pre-school, primary standards 1 to 6, primary standard 7, primary standard 8 or secondary forms 1 to 3, no female head/spouse, secondary form 4 or higher)* |
| **What kind of business (type of industry) is the main occupation of the male head/spouse connected with?** *(Does not work, no male head or spouse, agriculture, hunting, forestry, fishing, mining or quarrying, any other)* |
| **How many habitable rooms does this household occupy in its main dwelling (do not count bathrooms, toilets, storerooms, or garage)?** *(1, 2, 3, 4 or more)* |
| **How many mosquito nets does your household own?** *(None, 1, 2 or more)* |
| **How many towels does your household own?** *(None, one, two or more)* |
| **How many frying pans does your household own?** *(None, one, two or more)* |
| **Additional Grameen PPI – MOZAMBIQUE ONLY** |
| **Does the household have a clock (wall, wrist, or pocket)?** *(yes, no)* |
| **Does the household have a radio, stereo system, or cassette player?** *(No, radio only, stereo system or cassette player (regardless of radio))* |
| **Does the household have a bicycle, motorcycle, or car?** *(No, bicycle only, motorcycle or car (regardless of bicycle))* |
| **How many beds does the household have (single, double, bunk beds, or for children)?** *(None, one, two or more)* |
| **Additional Grameen PPI – THE GAMBIA ONLY** |
| **Does your household have a** **fridge**? *(yes, no)* |
| **Does your household have a** **television**? *(yes, no)* |
| **Does your household have a** **fan**? *(yes, no)* |
| **Can the** **female head of the household or the first wife read and write**? *(yes, no)* |
| **Socio-geographical variables** (from Tatenda Makanga) |
| **Are there** **neighbours or other families in your community who would help your household if there were pregnancy related problems?** *(yes, no, don’t know)* |
| 🡪 If yes, what form of help would your household receive? *(Transport, financial support, emotional or moral support, other, don’t know)* |
| **Is there a community group or organisation (formal or informal) that offers help to women who have problems during pregnancy?** *(yes, no, don’t know)* |
| 🡪 If yes, do you actively participate (e.g., attend meetings, contribute financially, activism) in this group or organization? *(yes, no, don’t know)* |
| **Who makes the decisions about money in your household?** *(husband, male partner, father, father-in-law, mother, mother-in-law, brother, brother-in-law, sister, sister-in-law, reproductive age woman, don’t know)* |
| **Who makes decisions about money related to pregnancy and pregnancy care?** *(husband, male partner, father, father-in-law, mother, mother-in-law, brother, brother-in-law, sister, sister-in-law, reproductive age woman, don’t know)* |
| **Do you have access to money/sufficient funds to arrange transport to seek care at the nearest facility?** *(yes, no, don’t know)* |

**Table S2C: Past history**

| **Obstetric history** |
| --- |
| **Have you ever given birth to a boy or girl who was born alive?** *(yes, no)* |
| 🡪 If yes, how many boys? *(number box)* |
| 🡪 If yes, how many girls? *(number box)* |
| 🡪 If yes, how many children do you have who are still alive? *(number box)* |
| **Have you ever had a pregnancy that miscarried or was aborted?** *(yes, no)* |
| 🡪 If yes, how many times? *(number box)* |
| **Have you ever given birth to a boy or girl who was stillborn?** *(yes, no)* |
| 🡪 If yes, how many in total? *(number box, don’t know)* |
| 🡪 If yes, how many boys? *(number box)* |
| 🡪 If yes, how many girls? *(number box)* |
| **Have you previously given birth to a boy or girl who was stillborn?** *(yes, no)* |
| Gravidity (calculated from live births + miscarriages + abortions + stillbirths) |
| Parity (calculated from stillbirths + livebirths) |
| 🡪 If at least one livebirth or stillbirth, did you ever give birth by Caesarean, that is, did they cut your belly open to take the baby out? *(yes, no)* |
| **Fertility/contraception** |
| **Have you used contraception in the last year?** *(yes, no, don’t know)* |
| 🡪 If yes, what method did you use? *(lactational amenorrhea, coitus interruptus, fertility awareness-based, barrier, injectable, subcutaneous rod, progestin-only pill, combined hormonal oral, emergency contraception, intrauterine, female and male sterilisation, other)* |
| 🡪 If no, would you have used contraception if it were available? *(yes, no, don’t know)* |
| **Have you received fertility treatment** **for this conception?** *(yes, no, don’t know)* |
| **Medical conditions diagnosed before pregnancy‡ (or in pregnancy if relevant)** |
| **Have you ever been told by a doctor or other health worker that you have high blood pressure or hypertension?** *(yes, no, don’t know)* |
| **Have you ever been put on a kidney machine?** *(yes, no, don’t know)* |
| **Have you ever been treated for “worms”?** *(yes, no, don’t know)* |
| **Have you ever been told by a doctor or other health worker that you have high blood sugar or diabetes?** *(yes, no, don’t know)* |
| **Have you ever been told by a doctor or other health worker that you have heart disease or a chronic heart condition?** *(yes, no, don’t know)* |
| **Have you ever had seizures** **unrelated to pregnancy/postpartum?** *(yes, no, don’t know)* |
| **Do you have chronic or active hepatitis?** *(yes, no, don’t know)* |
| **Do you have asthma?** *(yes, no, don’t know)* |
| **Do you have any other** **medical condition diagnosed before pregnancy?** *(yes, no, don’t know)* |
| 🡪 if yes, please provide further detail *(text box)* |

**Table S2D: Dietary Diversity Score screen**

| **Question: groups of foods (describe local examples)** |
| --- |
| **During the last 24 hours, have you eaten any foods made from grains, like: porridge, bread, rice, pasta/noodles, millet or other foods made from grains?** *(yes, no)* |
| **In the last month, how often have you eaten these foods?** *(every day, 2-4 times per week, 5-6 times per week, once a week, less than once a week)* |
| **During the last 24 hours, have you eaten any vegetables or root that are orange-coloured inside, like: pumpkin, carrots, squash or sweet potatoes that are yellow or orange inside?** *(yes, no)* |
| **In the last month, how often have you eaten these foods?** *(every day, 2-4 times per week, 5-6 times per week, once a week, less than once a week)* |
| **During the last 24 hours, have you eaten any white roots and tubers or plantains, such as: white potatoes, white yams, cassava/radish, or any other foods made from white-fleshed roots or tubers, or plantains?** *(yes, no)* |
| **In the last month, how often have you eaten these foods?** *(every day, 2-4 times per week, 5-6 times per week, once a week, less than once a week)* |
| **During the last 24 hours, have you eaten any dark green leafy vegetables, such as: List examples of any medium-to-dark green leafy vegetables, including wild-foraged leaves?** *(yes, no)* |
| **In the last month, how often have you eaten these foods?** *(every day, 2-4 times per week, 5-6 times per week, once a week, less than once a week)* |
| **During the last 24 hours, have you eaten any fruits that are dark yellow or orange inside, like: ripe mango, ripe papaya?** *(yes, no)* |
| **In the last month, how often have you eaten these foods?** *(every day, 2-4 times per week, 5-6 times per week, once a week, less than once a week)* |
| **During the last 24 hours, have you eaten any other vegetables: List examples of any other vegetables?** *(yes, no)* |
| **In the last month, how often have you eaten these foods?** *(every day, 2-4 times per week, 5-6 times per week, once a week, less than once a week)* |
| **During the last 24 hours, have you eaten any other fruits?** *(yes, no)* |
| **In the last month, how often have you eaten these foods?** *(every day, 2-4 times per week, 5-6 times per week, once a week, less than once a week)* |
| **During the last 24 hours, have you eaten any meat made from animal organs, such as: liver, kidney, heart or other organ meats or blood-based foods, including from wild game?** *(yes, no)* |
| **In the last month, how often have you eaten these foods?** *(every day, 2-4 times per week, 5-6 times per week, once a week, less than once a week)* |
| **During the last 24 hours, have you eaten any other types of meat or poultry, like: beef, pork, lamb, goat, rabbit, wild game meat, chicken, duck, other birds?** *(yes, no)* |
| **In the last month, how often have you eaten these foods?** *(every day, 2-4 times per week, 5-6 times per week, once a week, less than once a week)* |
| **During the last 24 hours, have you eaten any eggs: eggs from poultry or any other bird?** *(yes, no)* |
| **In the last month, how often have you eaten these foods?** *(every day, 2-4 times per week, 5-6 times per week, once a week, less than once a week)* |
| **During the last 24 hours, have you eaten any fish or seafood, whether fresh or dried: fresh or dried fish, shellfish or seafood?** *(yes, no)* |
| **In the last month, how often have you eaten these foods?** *(every day, 2-4 times per week, 5-6 times per week, once a week, less than once a week)* |
| **During the last 24 hours, have you eaten any beans or peas, such as: mature beans or peas (fresh or dried seed), lentils or bean/pea products, including hummus, tofu and tempeh?** *(yes, no)* |
| **In the last month, how often have you eaten these foods?** *(every day, 2-4 times per week, 5-6 times per week, once a week, less than once a week)* |
| **During the last 24 hours, have you eaten any nuts or seeds, like: any tree nut, ground nut, peanut or certain seeds or nut/seed “butters” or pastes?** *(yes, no)* |
| **In the last month, how often have you eaten these foods?** *(every day, 2-4 times per week, 5-6 times per week, once a week, less than once a week)* |
| **During the last 24 hours, have you eaten any milk or milk products such as: milk, cheese, yoghurt or other milk products but NOT including butter, ice cream, cream or sour cream?** *(yes, no)* |
| **In the last month, how often have you eaten these foods?** *(every day, 2-4 times per week, 5-6 times per week, once a week, less than once a week)* |
| **During the last 24 hours, have you eaten any condiments and seasonings, such as: condiments and seasonings: ingredients used in small quantities for flavour, such as chillies, spices, herbs, fish powder, tomato paste, flavour cubes or seeds?** *(yes, no)* |
| **In the last month, how often have you eaten these foods?** *(every day, 2-4 times per week, 5-6 times per week, once a week, less than once a week)* |
| **During the last 24 hours, have you eaten any freshly cooked fried snacks, like: samosa, wada, fritter?** *(yes, no)* |
| **In the last month, how often have you eaten these foods?** *(every day, 2-4 times per week, 5-6 times per week, once a week, less than once a week)* |
| **During the last 24 hours, have you eaten any savoury snacks in packets, like: potato crisps, Lays, kukure and other similar snacks?** *(yes, no)* |
| **In the last month, how often have you eaten these foods?** *(every day, 2-4 times per week, 5-6 times per week, once a week, less than once a week)* |
| **During the last 24 hours, have you eaten any bakery items, such as: cake, biscuits, puffs?** *(yes, no)* |
| **In the last month, how often have you eaten these foods?** *(every day, 2-4 times per week, 5-6 times per week, once a week, less than once a week)* |
| **During the last 24 hours, have you eaten any sweets?** *(yes, no)* |
| **In the last month, how often have you eaten these foods?** *(every day, 2-4 times per week, 5-6 times per week, once a week, less than once a week)* |
| **During the last 24 hours, have you eaten any fast food/street food/restaurant food?** *(yes, no)* |
| **In the last month, how often have you eaten these foods?** *(every day, 2-4 times per week, 5-6 times per week, once a week, less than once a week)* |
| **During the last 24 hours, have you consumed any fizzy drinks, such as: Coca Cola, Fanta, Limca?** *(yes, no)* |
| **In the last month, how often have you consumed these items?** *(every day, 2-4 times per week, 5-6 times per week, once a week, less than once a week)* |
| **During the last 24 hours, have you consumed any other drinks?** *(yes, no)* |
| **In the last month, how often have you consumed these items?** *(every day, 2-4 times per week, 5-6 times per week, once a week, less than once a week)* |

**Table S2E: Current pregnancy details**

| **Gestational age determination (1^st^ Visit Only)** |
| --- |
| **When did your/the woman’s last menstrual period (LMP) start?** *(yyyy/mm/dd)* |
| 🡪 Don’t know LMP start date? *(yes, no)* |
| **How certain are you/is she of this date?** *(1-Uncertain (ID=1), 2-Certain by menstrual cycle (ID=2), 3-Certain, by cycle and scan confirmed (ID=3), 4-Certain, by cycle, revised by scan)* |
| **Have you had an ultrasound scan in this pregnancy? (obstetric only – where available)** *(yes, no, don’t know)* |
| 🡪 if yes, what is the fetal sex? *(1 – Likely male (ID=1), 2- Likely female (ID=2), 3-Not seen (ID=3), 4-Not reported (ID=4))* |
| 🡪 If yes, what was the DATE of the **FIRST** scan? *(yyyy/mm/dd) – date will be used to determine whether u/s was <14 wks* |
| 🡪 DATE of the FIRST scan unknown? *(yes, no)* |
| 🡪 if yes, was an EDD assigned? *(yes, no, don’t know)* |
| 🡪 If yes, what EDD was assigned? *(yyyy/mm/dd)* |
| 🡪 EDD unknown? *(yes, no)* |
| 🡪 if yes, was a fetal abnormality detected? *(1-None (ID=1), 2-Not assessed (ID=2), 3-CNS (ID=3), 4-Cardiac (ID=4), 5-Urogenital (ID=5), 6-Gastrointestinal (ID=6), 7-Musculoskeletal (ID=7), 8-Respiratory (ID=8), 9-Multiple (ID=9), 10-Other Abnormality (ID=10), 11-Not visualised (ID=11))* |
| 🡪 if yes, what was the TCD (mm)? *(number box)* |
| 🡪 Don’t know TCD? *(yes, no)* |
| 🡪 if yes, what was the CRL (mm)? *(number box)* |
| 🡪 Don’t know CRL? *(yes, no)* |
| 🡪 if yes, what was the BPD (mm)? *(number box)* |
| 🡪 Don’t know BPD? *(yes, no)* |
| 🡪 if yes, what was the HC (mm)? *(number box)* |
| 🡪 Don’t know HC? *(yes, no)* |
| 🡪 if yes, what was the AC (mm)? *(number box)* |
| 🡪 Don’t know AC? *(yes, no)* |
| 🡪 if yes, what was the FL (mm)? *(number box)* |
| 🡪 Don’t know FL? *(yes, no)* |
| 🡪 if yes, is there a sub-chorionic haematoma? *(1=Yes, 0=No)* **Kenya and The Gambia only** |
| 🡪 if yes, is there a clinical suspicion of fetal growth restriction (FGR)? *(yes, no, don’t know) –* ***yes should trigger Table 4c - FGR*** |
| 🡪 if yes, is there a clinical indication for macrosomia? *(1= Yes, 0 = No)* |
| 🡪 if yes, what was the amniotic fluid index (AFI) (mm)? *(number box)* |
| 🡪 Don’t know AFI? *(yes, no)* |
| 🡪 if yes, what was the depth of the deepest amniotic fluid pocket (mm)? *(number box)* **Kenya and The Gambia only** |
| 🡪 Don’t know depth of deepest amniotic pocket? *(yes, no)* |
| 🡪 if yes, what was the umbilical RI? *(Text box)* **Kenya and The Gambia only** |
| 🡪 Don’t know umbilical RI? *(yes, no)* |
| 🡪 if yes, what was the umbilical PI? *(Text box)* **Kenya and The Gambia only** |
| 🡪 Don’t know umbilical PI? *(yes, no)* |
| 🡪 if yes, what was the umbilical EDV? (best value) *1-Present/intermittently absent (ID=1), 2-Absent/intermittently reversed (ID=2), 3-Reversed (ID=3), 4-No result (ID=4)* **Kenya and The Gambia only** |
| 🡪 Don’t know umbilical EDV? *(yes, no)* |
| 🡪 if yes, what was the L Ut RI? *(Text box)* **Kenya and The Gambia only** |
| 🡪 Don’t know L Ut RI? *(yes, no)* |
| 🡪 if yes, what was the R Ut RI? *(Text box)* |
| 🡪 Don’t know R Ut RI? *(yes, no)* |
| 🡪 if yes, what was the mean Ut RI? *(Text box; auto-calculated from two items above)* **Kenya and The Gambia only** |
| 🡪 Don’t know mean Ut RI? *(yes, no)* |
| 🡪 if yes, what was the L Ut PI? *(Text box)* **Kenya and The Gambia only** |
| 🡪 Don’t know L Ut PI? *(yes, no)* |
| 🡪 if yes, what was the R Ut PI? *(Text box)* **Kenya and The Gambia only** |
| 🡪 Don’t know R Ut PI? *(yes, no)* |
| 🡪 if yes, what was the mean Ut PI? *(Text box; calculated from two items above)* **Kenya and The Gambia only** |
| 🡪 Don’t know mean Ut PI? *(yes, no)* |
| 🡪 if yes, what was the L Notch? *1-Absent (ID=1), 2-Present (ID=2), 3-Indeterminate (ID=3), 4-Not visualized (ID=4), 5-No result (ID=5)* **Kenya and The Gambia only** |
| 🡪 Don’t know L Notch? *(yes, no)* |
| 🡪 if yes, what was the R Notch? *1-Absent (ID=1), 2-Present (ID=2), 3-Indeterminate (ID=3), 4-Not visualized (ID=4), 5-No result (ID=5)* **Kenya and The Gambia only** |
| 🡪 Don’t know R Notch? *(yes, no)* |
| **Has she had/Did she have a SECOND** **ultrasound?** *(yes, no, DK)* (**OPTIONS SHOULD BE SAME AS FOR FIRST ULTRASOUND)** |
| **Has she had/Did she have a THIRD ultrasound?** *(yes, no, DK)* (**OPTIONS SHOULD BE SAME AS FOR FIRST ULTRASOUND)** |
| **Has she had/Did she have a FOURTH ultrasound?** *(yes, no, DK)* (**OPTIONS SHOULD BE SAME AS FOR FIRST ULTRASOUND)** |
| Gestational age at consent **calculated** from date of visit and EDD, according to the following hierarchy: Ultrasound at <14wk, Ultrasound at ≥14 wk, Certain LMP (yyyy/mm/dd), Uncertain LMP (yyyy/mm/dd), SFH (SFH is asked under Fetal Assessment later in the visit) |
| **Other pregnancy characteristics** |
| **Does/did this baby have the same father as your other children?** *(no, yes, donor sperm, don’t know, not applicable (first pregnancy))* **1^st^ Visit Only** |
| **Early pregnancy complications** |
| **Have you had nausea or vomiting so far in this pregnancy?** *(yes, no, don’t know)* |
| **Did you have any vaginal bleeding in the first few months of your pregnancy/your most recent pregnancy?** *(yes, no, don’t know)* |
| 🡪 If yes, was it the size of a 1 dalasi piece (Gambia only); the size of the circle created by pressing your index finger to your thumb (Mozambique); the size of a 20 shilling coin (Kenya) *(yes, no, don’t know)* |
| **What position do you fall asleep in?** *(on back, on stomach, on side)* |
| **Antenatal care** |
| **Have you already received care for this pregnancy in this facility/Did you receive care for this most recent pregnancy in this facility?** *(yes, no) – this should be auto-populated from eligibility screen* |
| 🡪 If no, have you received care for this pregnancy at another facility/did you receive care for this most recent pregnancy at another facility? *(yes, no) - this should be auto-populated from eligibility screen* |
| 🡪 If yes, who advised you to attend that other facility? *(my partner/family, my health care provider, I decided myself, I decided with my partner/family)* |
| **Current vitamin or mineral supplements** |
| **Are you currently taking any vitamins or supplements?** *(yes, no, don’t know)* |
| 🡪 If yes, are you currently taking/did you take folic acid? *(yes, no, don’t know)* |
| 🡪 If yes, are you currently taking/did you take iron? *(yes, no, don’t know)* |
| 🡪 If yes, are you currently taking/did you take vitamin D? *(yes, no, don’t know)* |
| 🡪 If yes, are you currently taking/did you take calcium? *(yes, no, don’t know)* |
| 🡪 If yes, are you currently taking/did you take any other supplements? *(yes, no, don’t know)* |
| **Other medication** |
| **Are you currently taking any blood pressure (BP) medication?** *(yes, no, don’t know)* |
| 🡪 if yes, which BP medication are you currently taking/did you take? *(methyldopa, labetalol, nifedipine, other (specify), don’t know)* |
| **Are you currently taking any medication for diabetes?** *(yes, no, don’t know)* |
| 🡪 if yes, which diabetes medication are you currently taking/did you take? *(insulin, metformin, other oral hypoglycaemic agents (specify), don’t know)* |
| **Are you currently taking a thyroid supplement?** *(yes, no, don’t know)* |
| **Are you currently taking antibiotics?** *(yes, no, don’t know)* |
| **Are you currently taking antiretroviral therapy?** *(yes, no, don’t know)* |
| 🡪 if yes, please specify the type *(dual therapy, fixed-dose combination, other HAART, post-exposure prophylaxis, don’t know)* |
| **Are you currently taking an anti-seizure medication (not magnesium sulphate)?** *(yes, no, don’t know)* |
| **Are you currently taking aspirin?** *(yes, no, don’t know)* |
| 🡪 if yes, why are you taking aspirin/why did you take aspirin? *(headache, advised to take it, other reason (specify), don’t know)* |
| **Are you currently taking any other medication?** *(yes, no, don’t know) (text box) – antidepressant may come out here although women may not understand concept of depression* |
| 🡪 if yes, please specify *(Text box)* |
| 🡪 if yes, what is/was the medication for? *(text box)* |
| **Smoking (1^st^ Visit only)** |
| **Do you currently use tobacco products?** *(yes, no, do not wish to ask, do not wish to answer)* |
| 🡪 If yes, please specify which *(cigarettes, kreteks, pipes full of tobacco, cigars/cheroots/cigarillos, water pipe, snuff by mouth, snuff by nose, chewing tobacco, betel quid with tobacco, other (specify))* |
| 🡪 if cigarettes, on average, how many do you currently smoke each day? *(number box)* |
| 🡪 If other tobacco products used, how many times each day do you use these? *(number box)* |
| 🡪 if no, did you use any at all before pregnancy? *(yes, no, don’t know)* |
| 🡪 If no, do you live with people who smoke in your presence or are you regularly exposed to cigarette smoke in your workplace? *(no, yes, do not wish to answer)* |
| **Maternal clinical assessment (Every Visit)** |
| **What is the woman’s body weight** **today?** (kg) *(number boxes for 3 measurements – take three measurements in succession and we will record* ***median*** *weight)* |
| **What is the woman’s height today?** (cm) *(number box)* |
| **What is the woman’s sitting height?** (cm) *(number box)* |
| **What is the woman’s lower leg length?** (cm) *(number box)* |
| **What is the woman’s mid-upper arm circumference?** (cm) *(number box)* |
| **What is the woman’s respiratory rate?** (breaths/min based on 30 sec measurement) *(number box)* |
| 🡪 Not available? *(yes, no)* |
| **What is the woman’s pulse** **rate?** (from CRADLE device - bpm) *(number box)* |
| 🡪 Not available? *(yes, no)* |
| **What is the woman’s pulse oximetry?** (%) *(number box)* |
| 🡪 Not available? *(yes, no)* |
| **What is the woman’s haemoglobin concentration?** (%) *(number box)* |
| 🡪 Not available? *(yes, no)* |
| **What is/was the woman’s perfusion index?** *(number box)* |
| 🡪 Not available? *(yes, no)* |
| **What is the woman’s carboxyhaemoglobin saturation? (SPOC)** *(number box)* |
| 🡪 Not available? *(yes, no)* |
| **What is the woman’s resting systolic BP?** (mmHg) *(number box)* 🡪 **if sBP≥140 or dBP≥90, eligible for ‘ToD hypertension’ cohort, perhaps after entering proteinuria data?** *(number box)* |
| 🡪 Not available? *(yes, no)* |
| **What is the woman’s resting diastolic BP?** (mmHg) *(number box/number box, DK, not available)* **🡪 if sBP≥140 or dBP≥90, eligible for ‘ToD hypertension’ cohort, perhaps after entering proteinuria data?** *(number box)* |
| 🡪 Not available? *(yes, no)* |
| **Was dipstick urinalysis for proteinuria done?** *(yes, no, don’t know)* |
| 🡪 if yes, what was the result? *(1-Neg/trace (ID=1), 2-1+ or 0.3 g/L (ID=2), 3-2+ or 1 g/L (ID=3), 4-3+ or >=3 g/L (ID=4), 5-Not done (ID=5))* |
| **Was dipstick urinalysis of urinary** **nitrites done?**  *(yes, no, don’t know)* |
| 🡪 if yes, what was the result? *(none, 1+, 2+, 3+)* |
| **Was dipstick urinalysis for leukocytes done?** *(yes, no, don’t know)* |
| 🡪 if yes, what was the result? *(none, 1+, 2+, 3+)* |
| **Was dipstick urinalysis for glucose done?** *(yes, no, don’t know)* |
| 🡪 if yes, what was the result? *(none, 1+, 2+, 3+)* |
| 🡪 if ≥1+, was a clinical diagnosis of GDM made? *(yes, no, don’t know)* |
| **Fetal assessment** |
| **Within the last 24 hours, have you felt decreased fetal movement?** *(yes, no, don’t know)* |
| 🡪 if yes, when were fetal movements last felt? *(hh/mm, don’t know)* |
| 🡪 Don’t know the time of fetal movements? *(yes, no)* |
| **Is/was the fetal heart rate (FHR) present or absent?** *(present, absent, not done, don’t know)* |
| 🡪 if absent, DATE on which FHR was confirmed to be absent? *(today or yyyy/mm/dd)* **eligible for ‘ToD IUFD/stillbirth’ cohort** |
| 🡪 DATE Don’t know? *(yes, no)* |
| 🡪 if absent, TIME at which FHR was confirmed to be absent? *(hh/mm)* |
| 🡪 TIME Don’t know? *(yes, no)* |
| 🡪 if absent, how was FHR measured? *(Pinard, Doptone, Cardiotocography, don’t know)* |
| **What is the SFH?** *(cm)* *(number box)*  🡪 if 16-36 weeks, and measurement ≤2cm than GA (wks), **eligible for ‘ToD FGR’ cohort** |
| 🡪 Don’t know SFH? *(yes, no)* |
| **Prenatal screening* (1^st^ visit only)** |
| **Has the woman previously been screened for Rhesus status?** *(yes, no, don’t know)* |
| **Has the woman previously been screened for HIV in this pregnancy?** *(yes, no, don’t know)* |
| **Has the woman previously been screened for TB in this pregnancy?** *(yes, no, don’t know)* |
| 🡪 if yes, which test(s) were used? *(skin test, IGRA)* |
| **Has the woman previously been screened for malaria in this pregnancy?** *(yes, no, don’t know)* |
| 🡪 if yes, which test was used? *(blood film, RDT, PCR, other (specify), DK)* |
| **Has the woman previously been screened for syphilis in this pregnancy?** *(yes, no, don’t know)* |
| **Were the woman’s stools previously tested for ova and parasites in this pregnancy**? *(yes, no, don’t know)* |
| **Investigations (Up to and including this visit if it is the 1^st^ visit, or since last study assessment if this is the 2^nd^ ANC visit since consent)** |
| **Has the woman’s haemoglobin concentration been measured in this pregnancy to date?/Has the woman’s haemoglobin concentration been measured since the last PRECISE visit?** (hemocue) *(no, yes, not tested, unavailable, don’t know)* |
| **Has the woman’s white cell count been** **tested in this pregnancy to date?/ Has the woman’s white cell count been** **tested since the last PRECISE visit?** *(no, yes, not tested, unavailable, don’t know)* |
| **Has the woman’s platelet count been tested in this pregnancy to date?/Has the woman’s platelet count been tested since the last PRECISE visit** *(no, yes, not tested, unavailable, don’t know)* |
| **Has the woman’s serum creatinine level been tested in this pregnancy to date?/Has the woman’s serum creatinine level been tested since the last PRECISE visit?** *(no, yes, not tested, unavailable, don’t know)* |
| **Has the woman’s aspartate transaminase level been tested in this pregnancy to date?/Has the woman’s aspartate transaminase level been tested since the last PRECISE visit?** *(no, yes, not tested, unavailable, don’t know)* |
| **Has the woman’s alanine aminotransferase level been tested in this pregnancy to date?/Has the woman’s alanine aminotransferase level been tested since the last PRECISE VISIT?** *(no, yes, not tested, unavailable, don’t know)* |
| **Visit Summary** |
| **Was the woman admitted to antenatal ward for monitoring and-or treatment?** *(yes, no)* |
| **Was the woman admitted to labour ward?** *(yes, no)* |
| **Was the woman admitted to maternity waiting home?** *(yes, no)* |
| **Was the woman referred to another service within facility?** *(yes, no)* |
| **Was the woman referred to another facility?** *(yes, no)* |
| **Sample Collection** |
| **Was maternal blood collected?** *(yes, no)* |
| **🡪** if no, why? *(no consent, participant refused, no staff member available to collect sample, no staff member available to process the sample, research team were unable to successfully draw blood, participant left before sample was collected, other (specify), don’t know)* |
| **Was maternal urine collected?** *(yes, no)* |
| **🡪** if no, why? *(no consent, participant refused, no staff member available to collect sample, no staff member available to process the sample, participant unable to produce sample, other (specify), don’t know)* |
| **Was a vaginal swab collected?** *(yes, no)* |
| **🡪** if no, why? *(no consent, participant refused, no staff member available to collect sample, no staff member available to process the sample, other (specify), don’t know)* |

**Table S2F: Additional questions for MMWG to be asked only at the 2^nd^ PRECISE visit, in the third trimester**

| **Maternal symptoms experienced during the past 2 weeks** |
| --- |
| **In the past 2 weeks, have you had red, inflamed or bleeding gums?** *(yes, no)* |
| **In the past 2 weeks, have you had chest pain?** *(yes, no)* |
| **In the past 2 weeks, have you felt lightheaded?** *(yes, no)* |
| **In the past 2 weeks, have you had decreased exercise tolerance?** *(yes, no)* |
| **In the past 2 weeks, has your heart been beating very fast or too fast (palpitations)?** *(yes, no)* |
| **In the past 2 weeks, have you been breathing faster than usual?** *(yes, no)* |
| **In the past 2 weeks, have you had difficulty breathing?** *(yes, no)* |
| **In the past 2 weeks, have you lost any teeth?** *(yes, no)* |
| **WHODAS** |
| **In the past 30 days, how much difficulty did you have in standing for long periods such as 30 minutes?** (*None, mild, moderate, severe, extreme or cannot do)* |
| **In the past 30 days, how much difficulty did you have in taking care of household responsibilities?** (*None, mild, moderate, severe, extreme or cannot do)* |
| **In the past 30 days, how much difficulty did you have in learning a new task, for example, learning how to get to a new place?** (*None, mild, moderate, severe, extreme or cannot do)* |
| **In the past 30 days, how much of a problem did you have joining in community activities (for example, festivities, religious or other activities) in the same way as anyone else can?** (*None, mild, moderate, severe, extreme or cannot do)* |
| **In the past 30 days, how much have you been emotionally affected by your health problems?** (*None, mild, moderate, severe, extreme or cannot do)* |
| **In the past 30 days, how much difficulty did you have in concentrating on doing something for ten minutes?** (*None, mild, moderate, severe, extreme or cannot do)* |
| **In the past 30 days, how much difficulty did you have in walking a long distance such as a kilometre [or equivalent]?** (*None, mild, moderate, severe, extreme or cannot do)* |
| **In the past 30 days, how much difficulty did you have in washing your whole body?** (*None, mild, moderate, severe, extreme or cannot do)* |
| **In the past 30 days, how much difficulty did you have in getting dressed?** (*None, mild, moderate, severe, extreme or cannot do)* |
| **In the past 30 days, how much difficulty did you have in dealing with people you do not know?** (*None, mild, moderate, severe, extreme or cannot do)* |
| **In the past 30 days, how much difficulty did you have in maintaining a friendship?** (*None, mild, moderate, severe, extreme or cannot do)* |
| **In the past 30 days, how much difficulty did you have in your day-to-day work/school?** (*None, mild, moderate, severe, extreme or cannot do)* |
| **Overall, in the past 30 days, how many days were these difficulties present?** (Record number of days) |
| **In the past 30 days, for how many days were you totally unable to carry out your usual activities or work because of any health condition?** (Record number of days) |
| **In the past 30 days, not counting the days that you were totally unable, for how many days did you cut back or reduce your usual activities or work because of any *health* condition?** (Record number of days) |
| **In the past 30 days, how would you rate your overall health?** *(Very good, good, neither poor nor good, poor, very poor)* |

**Table S2G: ToD pregnancy**

| **Maternal symptoms** (within the last 24hr) |
| --- |
| **Within the last 24 hrs, have you had a headache?** *(yes, no)* |
| **Within the last 24 hrs, have you had visual symptoms** *(yes, no)* |
| **Within the last 24 hrs, have you had chest pain?** *(yes, no)* |
| **Within the last 24 hrs, have you experienced shortness of breath?** *(yes, no)* |
| **Within the last 24 hrs, have you had any abdominal pain?** *(yes, no)* |
| **Within the last 24 hrs, have you had any vaginal bleeding?** *(yes, no)* |
| 🡪 if yes, how big was the blood spot? *(size of a 1 dalasi piece (The Gambia); size of a circle formed with your thumb and index finger (Mozambique); size of a 20 shilling coin (Kenya))* |
| **Lab** |
| **What is the woman’s pulse oximetry?** (%) *(number box)* |
| 🡪 Don’t know pulse oximetry? *(yes, no)* |
| **Were blood tests requested?** *(yes, no)* |
| 🡪 if yes, was the woman’s **haemoglobin** level tested? (hemocue) *(no – test not required, no - test not available, yes)* |
| 🡪 if yes, was the woman’s **WCC** tested? *(no – test not required, no - test not available, yes)* |
| 🡪 if yes, was the woman’s **platelet count** tested? *(no – test not required, no - test not available, yes)* |
| 🡪 if yes, was the woman’s **serum creatinine** tested? *(no – test not required, no - test not available, yes)* |
| 🡪 if yes, was the woman’s **AST** tested? *(no – test not required, no - test not available, yes)* |
| 🡪 if yes, was the woman’s **ALT** tested? *(no – test not required, no - test not available, yes)* |
| **Was any other investigation(s)† carried out?** *(yes, no)* |
| **Fetal assessment** |
| **Was an ultrasound done following the diagnosis of hypertension, suspected fetal growth restriction (FGR), or interuterine fetal death (IUFD)?** *(yes, no, don’t know)* |
| 🡪 If yes, what was the DATE of the ultrasound? *(yyyy/mm/dd)* |
| 🡪 Don’t know the date of ultrasound? *(yes, no)* |
| 🡪 if yes, was a fetal abnormality diagnosed? *(yes, no)* |
| 🡪 if yes, specify type *(1-None (ID=1), 2-Not assessed (ID=2), 3-CNS (ID=3), 4-Cardiac (ID=4), 5-Urogenital (ID=5), 6-Gastrointestinal (ID=6), 7-Musculoskeletal (ID=7), 8-Respiratory (ID=8), 9-Multiple (ID=9), 10-Other Abnormality (ID=10), 11-Not visualised (ID=11))* |
| 🡪 If yes, what was the TCD? (mm) *(number box, don’t know)* |
| 🡪 Don’t know TCD? *(yes, no)* |
| 🡪 If yes, what was the CRL? (mm) *(number box, don’t know)* |
| 🡪 Don’t know CRL? *(yes, no)* |
| 🡪 If yes, what was the BPD? (mm) *(number box, don’t know)* |
| 🡪 Don’t know BPD? *(yes, no)* |
| 🡪 If yes, what was the HC? (mm) *(number box, don’t know)* |
| 🡪 Don’t know HC? *(yes, no)* |
| 🡪 If yes, what was the AC? (mm) *(number box, don’t know)* |
| 🡪 Don’t know AC? *(yes, no)* |
| 🡪 If yes, what was the FL? (mm) *(number box, don’t know)* |
| 🡪 Don’t know FL? *(yes, no)* |
| 🡪 if yes, is there a clinical suspicion of FGR? *(yes, no)* |
| 🡪 if yes, what was the AFI (mm)? *(Text box)* |
| 🡪 Don’t know AFI? *(yes, no)* |
| 🡪 if yes, what was the depth of the deepest amniotic fluid pocket (mm) *(Text box)* **Kenya and The Gambia only** |
| 🡪 Don’t know depth of the deepest amniotic fluid pocket? *(yes, no)* |
| 🡪 if yes, what was the umbilical RI *(Text box)* **Kenya and The Gambia only** |
| 🡪 Don’t know umbilical RI? *(yes, no)* |
| 🡪 if yes, what was the umbilical PI *(Text box)* **Kenya and The Gambia only** |
| 🡪 Don’t know umbilical PI? *(yes, no)* |
| 🡪 if yes, what was the umbilical EDV *1-Present/intermittently absent (ID=1), 2-Absent/intermittently reversed (ID=2), 3-Reversed (ID=3), 4-No result (ID=4)* **Kenya and The Gambia only** |
| 🡪 Don’t know umbilical EDV? *(yes, no)* |
| 🡪 if yes, what was the L Ut RI *(Text box)* **Kenya and The Gambia only** |
| 🡪 Don’t know L Ut RI? *(yes, no)* |
| 🡪 if yes, what was the R Ut RI *(Text box)* **Kenya and The Gambia only** |
| 🡪 Don’t know R Ut RI? *(yes, no)* |
| 🡪 if yes, what was the mean Ut RI *(Text box; calculated from two previous items)* **Kenya and The Gambia only** (autocalculated) |
| 🡪 if yes, what was the L Ut PI *(Text box)* **Kenya and The Gambia only** |
| 🡪 Don’t know L Ut PI? *(yes, no)* |
| 🡪 if yes, what was the R Ut PI *(Text box)* **Kenya and The Gambia only** |
| 🡪 Don’t know R Ut PI? *(yes, no)* |
| 🡪 if yes, what was the mean Ut PI *(Text box; calculated from two previous items)* **Kenya and The Gambia only** (autocalculated) |

**Table S2H: Delivery suite module**

| **Date and time of delivery** |
| --- |
| **What was the location of the delivery?** *(home, PHC, district hospital, regional hospital, tertiary hospital, private hospital/clinic, en route, other, don’t know)* |
| 🡪 if the delivery was at a PHC, which PHC? *(Kenya – Rabai, Moz – Xinavane, The Gambia – Sara Kunda, Illiasa, Ngenyen Sanjal, Njaba Kunda, other, don’t know)* |
| 🡪 if other, specify *(text box)* |
| 🡪 if the delivery was at a district hospital, which district hospital? *(Kenya – Mariakani, Moz – Manhica, The Gambia – Farafenni, other, don’t know)* |
| 🡪 if other, specify *(text box)* |
| **What was the DATE of arrival at the facility for delivery admission?** *(yyyy/mm/dd, don’t know, N/A)* |
| **What was the TIME of arrival at the facility for delivery admission?** *(timebox on 2400 clock, don’t know, N/A)* |
| **What was the DATE of first assessment at the facility for delivery admission?** *(yyyy/mm/dd, don’t know, N/A)* **(Facility delivery only)** |
| **What was the TIME of first assessment at the facility for delivery admission?** *(timebox on 2400 clock, don’t know, N/A)* **(Facility delivery only)** |
| **Antenatal Care** |
| **Did the woman have antenatal care prior to this delivery admission?** *(yes, no, don’t know)* |
| 🡪 If yes, how many times did she receive antenatal care during this pregnancy? *(number box)* |
| 🡪 If yes, as part of her antenatal care was her BP measured at least once during the pregnancy? *(yes, no, don’t know)* |
| 🡪 If yes, was the woman’s BP measured at EVERY visit? *(yes, no, don’t know)* |
| **During this pregnancy, were you given an injection in the arm to prevent the baby from getting tetanus, that is, convulsions after birth?** *(yes, no)* |
| **During this pregnancy, were you given a tablet to prevent malaria?** *(textbox for number of times, don’t know)* |
| 🡪 If yes, how many times were you given this tablet? *(text box for number of times, don’t know)* |
| **Was an insecticide-treated mosquito net issued**? *(yes, no)* |
| 🡪 If yes, did you sleep under this net? *(yes, no, don’t know)* |
| **Was the woman screened for GDM?** *(yes, no, don’t know)* |
| 🡪 if yes, did she receive a clinical diagnosis of GDM? *(yes, no, don’t know)* |
| 🡪 if yes, how was this treated? *(diet, medication, not treated, don’t know)* |
| **Fetal assessment on admission** *for women not known to have a stillbirth* |
| **Was the fetal heart rate measured on admission to the facility?** *(yes, no, don’t know, N/A)* |
| 🡪 If yes, was the FHR heard (FHR activity confirmed)? *(yes, no, don’t know)* |
| 🡪 if no, go to **ToD IUFD/stillbirth cohort** |
| 🡪 If yes, what was used? *(Pinard, Doptone, cardiotograph, don’t know)* |
| **Investigations – for women who were admitted to PRECISE facilities only** |
| **Was the woman’s haemoglobin concentration measured?** (hemocue) *(yes, no, unavailable, not tested, don’t know, N/A)* |
| **Was the woman’s WCC** **tested?** *(yes, no, unavailable, not tested, don’t know, N/A)* |
| **Was the woman’s platelet count tested?** *(yes, no, don’t know, N/A)* |
| **Was the woman’s serum creatinine level tested?** *(yes, no, don’t know, N/A)* |
| **Was the woman’s aspartate transaminase tested?** *(yes, no, don’t know, N/A)* |
| **Was the woman’s alanine aminotransferase tested?** *(yes, no, don’t know, N/A)* |
| **The delivery** |
| **Please indicate the number of baby(ies) in this pregnancy *(number box)*** |
| **Was an attendant present at the delivery?** *(yes, no, don’t know)* |
| 🡪 if yes, who attended the delivery? *(doctor, midwife, nurse, community health workers, traditional birth attendant, family member, none) – please select all that apply* |
| **How was the delivery initiated?** *(spontaneous onset of labour, caregiver-initiated by labour induction, caregiver-initiated by elective Caesarean, caregiver-initiated by emergency Caesarean, caregiver-initiated by termination of pregnancy, woman died undelivered, don’t know)* |
| 🡪 If caregiver-initiated by any method, what was the indication? Please select all that apply *(fetal indication (specify), maternal indication (including. trauma†, specify), fetal AND maternal indication, unknown)* |
| 🡪 If labour was induced, what method(s) was used? *(Please select all that apply: vaginal prostaglandins, balloon catheter, oral misoprostol, vaginal misoprostol, amniotomy, intravenous oxytocin, other (specify))* |
| **Maternal monitoring BEFORE birth** |
| **During the admission and before birth, was the woman’s BP checked?** *(yes, no, don’t know)* |
| 🡪 if yes, what was the highest sBP? *(number box, don’t know)* |
| 🡪 if yes, what was the highest dBP? *(number box, don’t know)* |
| 🡪 if yes, what was the lowest sBP? *(number box, don’t know)* |
| 🡪 if yes, what was the lowest dBP? *(number box, don’t know)* |
| **During the admission and before birth, was the woman’s urinary protein checked?** *(yes, no, don’t know)* |
| 🡪 if yes, what was the highest documented level of proteinuria for each of the following methods: urinary dipstick *(Neg/trace, +1, +2, +3),* protein:creatinine ratio (in g/mol), 24hr urinary protein (g/d) |
| **During the admission and before birth, was the mother’s temperature checked?** *(yes, no, don’t know)* |
| 🡪 if yes, what was the highest maternal temperature? *(C or F, text box, don’t know)* |
| 🡪 if yes, what was the lowest maternal temperature? *(C or F, text box, don’t know)* |
| **During the admission and before birth, was the mother’s HR checked?** *(yes, no, don’t know)* |
| 🡪 if yes, what was the highest HR? *(number box, don’t know)* |
| **During the admission and before birth, was the mother’s RR checked?** *(yes, no, don’t know)* |
| 🡪 if yes, what was the highest RR? *(number box, don’t know)* |
| 🡪 if yes, what was the lowest RR? *(number box, don’t know)* |
| **During the admission and before birth, was the mother’s oxygen saturation checked?** *(yes, no, don’t know)* |
| 🡪 if yes, what was the lowest oxygen saturation? *(number box, don’t know)* |
| 🡪 if yes, was the oxygen saturation <90% for at least 60 min? *(yes, no, don’t know)* |
| **During the admission and before birth, was the woman’s haemoglobin concentration checked?** *(yes, no, don’t know)* |
| 🡪 if yes, go to the Lab and Test results tab to record the result |
| **Maternal monitoring AFTER birth** |
| **After birth, was the woman’s BP checked?** *(yes, no, don’t know)* |
| 🡪 if yes, what was the highest sBP? *(number box, don’t know)* |
| 🡪 if yes, what was the highest dBP? *(number box, don’t know)* |
| 🡪 if yes, what was the lowest sBP? *(number box, don’t know)* |
| 🡪 if yes, what was the lowest dBP? *(number box, don’t know)* |
| **After birth, was the woman’s temperature checked?** *(yes, no, don’t know)* |
| 🡪 if yes, what was the highest temperature recorded? *(number box, don’t know)* |
| 🡪 if yes, what was the lowest temperature recorded? *(number box, don’t know)* |
| **After birth, was the woman’s HR checked?** *(yes, no, don’t know)* |
| 🡪 if yes, what was the highest HR? *(number box, don’t know)* |
| **After birth, was the maternal RR checked?** *(yes, no, don’t know)* |
| 🡪 if yes, what was the highest RR? *(number box, don’t know)* |
| 🡪 if yes, what was the lowest RR? *(number box, don’t know)* |
| **After delivery, was the woman’s oxygen saturation checked?** *(yes, no, don’t know)* |
| 🡪 if yes, what was the lowest oxygen saturation? *(number box, don’t know)* |
| 🡪 if yes, was the oxygen saturation <90% for at least 60 min? *(yes, no, don’t know)* |
| **Membrane rupture** |
| **Did the membranes rupture prior to delivery (or maternal death if undelivered)?** *(yes, no, don’t know)* |
| 🡪 If yes, how did the membranes rupture? *(spontaneously, caregiver-initiated, don’t know)* |
| 🡪 if caregiver-initiated per vaginam, what was the indication? *(for prevention of delay in labour, to augment labour, to induce labour, other (please specify))* |
| 🡪 if yes, what was the DATE on which the membranes ruptured? *(date box yyyy/mm/dd, don’t know)* |
| 🡪 if yes, what was the TIME at which the membranes ruptured? *(time box 2400 clock, don’t know)* |
| 🡪 if DK, how much time passed between membrane rupture and delivery? *(<12 hour, 12-24 hours, >24 hours, don’t know)* |
| 🡪 if yes, was there meconium-staining of the amniotic fluid? *(yes, no, don’t know)* |
| 🡪 if yes, was the amniotic fluid offensive in odour? *(yes, no, don’t know)* |
| **FGM** |
| **Does the woman present with FGM?** *(no/yes but type uncertain/yes, Type 1 clitoridectomy/yes, Type 2 excision/yes, Type 3 infibulation/yes, Type 4 all other harmful procedures, don’t know)* |
| **Interventions** |
| **Did the woman have a cervical cerclage?** *(yes, no, don’t know)* |
| 🡪 if yes, what was the date of insertion? (to calculate GA at insertion) *(yyyy/mm/dd or don’t know)* |
| 🡪 if yes, what was the date of removal? (to calculate GA at removal) *(yyyy/mm/dd or don’t know)* |
| **The placenta & uterus** |
| **Did the woman have placenta praevia?** (implantation of the placenta over the internal os of the cervix, as identified by ultrasound scan, vaginal examination or at time of delivery) *(yes, no, don’t know)* |
| **Did the woman have placental abruption?** (premature separation of the placenta from the uterine wall, that is diagnosed clinically, usually by a combination of vaginal bleeding, maternal abdominal pain, and retroplacental blood clots at delivery) *(yes, no, don’t know)* |
| **Did the woman have a fibroid uterus?** *(yes, no, don’t know)* |
| **Labour** |
| **Did labour occur?** *(yes, no, don’t know)* |
| 🡪 If yes, was the FHR monitored during labour? *(yes, no, don’t know)* |
| 🡪 If yes, what was used? *(Pinard, Doptone, CTG, don’t know)* |
| 🡪 if cardiotocograph, was this performed continuously? *(yes, no, don’t know)* |
| 🡪 if yes, was a partogram used? *(yes, no, don’t know, N/A)* |
| 🡪 If yes, at how dilated was the woman’s cervix when the partogram was commenced? *(dropdown options 0 to 10 cm)* |
| 🡪 if yes, was there vaginal bleeding before delivery? *(yes, no, don’t know)* |
| 🡪 if yes, what was the DATE and TIME of onset of  1^st^ stage of labour (regular painful contractions as per the woman)? ‡ *(dd/mmm/yyyy and 2400) (date box and timebox or don’t know)* |
| 🡪 if yes, what was the DATE and TIME of onset of 2^nd^ stage of labour (from full dilatation)? *(yyyy/mm/dd and 2400) (date box and time box)* |
| 🡪 if yes, what was the DATE and TIME of delivery of LAST baby? *(yyyy/mm/dd and 2400 or don’t know) (duration of 2^nd^ stage calculated from the DATE and TIME of delivery of the LAST baby to the DATE and TIME of delivery of the LAST placenta)* |
| 🡪 if don’t know, what was the duration of labour? *(<12hr, 12-24hr, >24hr, don’t know)* |
| 🡪 if yes, what was the DATE and TIME of delivery of LAST placenta? *(yyyy/mm/dd and 2400 or don’t know) (duration of 3^rd^ stage calculated from the DATE and TIME of delivery of LAST baby to DATE and TIME of delivery of LAST placenta)* |
| 🡪 if don’t know, how long did it take to deliver the placenta? *(<10min, 10-30min, >30min, DK)* |
| 🡪 if yes, was labour augmented? *(yes, no, don’t know, N/A)* |
| 🡪 if yes, how was it augmented? *(ARM, oxytocin, misoprostol, other, don’t know)* |
| 🡪 if yes, at what stage was augmentation initiated? *(<5cm dilatation, >=5cm dilatation, don’t know)* |
| 🡪 if yes, did the woman receive any form of pain relief? *(epidural analgesia, opioids, relaxation techniques, massage, water, nitrous oxide, other (please specify))* |
| 🡪 if yes, what positions was the woman in during labour? *(tick all that apply: supine, semi-recumbent, standing, walking, squatting, all-fours, other (please specify), don’t know)* |
| 🡪 did the woman proceed to giving birth vaginally? *(yes, no, don’t know)* |
| **Was an episiotomy performed?** *(yes, no, don’t know)* |
| 🡪 if yes, what was the indication? *(fetal distress, shoulder dystocia, other)* |
| **Did the woman experience a cervical or perineal laceration?** *(no, yes – 1^st^ to 2^nd^ degree, yes – 3^rd^ to 4^th^ degree, cervical laceration, don’t know)* |
| **Care in third stage** |
| **Were prophylactic uterotonics administered?** *(yes, no, don’t know)* |
| 🡪 If yes, which uterotonics were administered? Tick all that apply *(oxytocin, ergometrine, misoprostol, other (specify), don’t know)* |
| 🡪 If misoprostol was administered, how was it administered? *(orally, vaginally, rectally, don’t know)* |
| **Management during delivery admission** (antepartum/postpartum) |
| **If the woman is HIV positive, was ART initiated?** *(no/yes at diagnosis/yes only for labour and delivery, don’t know)* |
| **Was antihypertensive treatment provided before or after birth?** *(yes, no, don’t know)* |
| 🡪 if yes, what type of antihypertensive medication was prescribed? (Please select ALL that apply.) *(hydralazine, labetalol, nifedipine, methyldopa, other)* |
| **Was magnesium sulphate treatment provided before or after birth?** *(yes, no, don’t know)* |
| 🡪 if yes, what was the indication(s)? (Please select ALL that apply.) *(pre-eclampsia, eclampsia, fetal neuroprotection (preterm delivery), don’t know)* |
| **Were steroids administered prior to delivery?** *(yes, no, don’t know)* |
| 🡪 if yes, why? *(fetal lung maturation, other indication, don’t know)* |
| **Were antibiotics administered to the mother before or after birth?** *(yes, no, don’t know)* |
| 🡪 if yes, why? *(prolonged rupture of membranes, C-section, Group B strep infection, maternal sepsis, raised temperature during labour, other indication (please specify), don’t know)* |
| 🡪 if yes, what type of antibiotic was prescribed? (Please select ALL that apply.) *(first generation cephalosporin, penicillin, other (specify)* |

**Table S2I: Maternal outcomes and care**

|  |
| --- |
| **Maternal death** |
| **Did the woman die?** *(yes, no)* |
| 🡪 if she died, what was the **DATE** of death? *(yyyy/mm/dd)* |
| 🡪 if she died, what was the **TIME** of death? *(hh/mmm)* |
| 🡪 if she died, **WHERE** did she die? *(home, PHC, district hospital, regional hospital, tertiary hospital, private hospital/clinic, en route, other, don’t know)* |
| 🡪 if she died at a PHC, **WHICH** PHC did she die at? *(Kenya – Rabai; Moz – Maragra, Malavele, Palmeiras, Três de Fevereiro, Taninga, Ilha Josina, Maluana, Munguine; The Gambia – Sara Kunda, Illiasa, Ngenyen Sanjal, Njaba Kunda, other, don’t know)* |
| 🡪 if she died at a district hospital, **WHICH** district hospital did she die at? *(Kenya – Mariakani; Moz –Xinavane, Manhiça; The Gambia – Farafenni, other, don’t know)* |
| 🡪 (unless death was at home or don’t know), what was the woman’s status on arrival at facility? *(alive and not critically ill, alive and critically ill, dead on arrival, don’t know)* |
| 🡪 (unless death was at home or don’t know), had the woman been referred from another centre? *(yes, no, don’t know)* |
| 🡪 if yes, what was the reason for her referral? *(high blood pressure, pre-eclampsia, eclampsia, obstetric haemorrhage before or after delivery, serious infection, prolonged labour, other🡪 please specify)* |
| 🡪 if she died, what was the **timing relative to birth**? *(before birth, during labour and delivery, after birth, don’t know)* |
| 🡪 If she died, please provide a case **summary** (short summary of the sequence of events surrounding the death) *(text box)* |
| 🡪 if she died, was an **autopsy** performed? *(yes, no, don’t know)* |
| 🡪 if she had an autopsy, please provide details *(summary of case [text box] or not available)* |
| 🡪 if she had an autopsy, what was the cause of death by ICD-MM group? *(1=Pregnancies with abortive outcome, 2=hypertensive disorders in pregnancy, childbirth and the puerperium, 3=obstetric haemorrhage, 4=pregnancy-related infection, 5=other obstetric complications (includes ectopic, ruptured uterus, obstructed labour), 6=unanticipated complications of management (medical/surgical area), 7=non-obstetric complications (cardiac disease, endocrine conditions, GI tract conditions, CNS, GU, autoimmune disorders, skeletal, psychiatric, neoplasms, infections that are not a direct result of pregnancy), 8=unknown/undetermined, 9=co-incidental causes (due to external causes)) these would be collected monthly by the central team* |
| 🡪 if 2, did the woman receive antihypertensive drugs for sBP ≥160 or dBP ≥110mmHg? *(yes, no, don’t know)* |
| 🡪 if she did not have an autopsy, what was the **cause of death** according to the care providers? (*text box*) – review by central team to assign ICD-MM group: *1=Pregnancies with abortive outcome, 2=hypertensive disorders in pregnancy, childbirth and the puerperium, 3=obstetric haemorrhage, 4=pregnancy-related infection, 5=other obstetric complications (includes ectopic, ruptured uterus, obstructed labour), 6=unanticipated complications of management (medical/surgical area), 7=non-obstetric complications (cardiac disease, endocrine conditions, GI tract conditions, CNS, GU, autoimmune disorders, skeletal, psychiatric, neoplasms, infections that are not a direct result of pregnancy), 8=unknown/undetermined, 9=co-incidental causes (due to external causes))* |
| 🡪 if she died, did any of the following **personal or family factors** contribute to the death of this woman? *(delay in seeking help, failure to recognise danger signs, declined treatment or admission, ignorance of available services, cultural or religious reasons or objections, poverty, other personal or family factors, don’t know, no)* |
| 🡪 if she died, did any of the following **transport/communication factors** contribute to the death of this woman? *(*(*lack of transport from home to facility, lack of transport between health facilities, health service communication breakdown, long distance, poor roads, other, no)* |
| 🡪 if she died, did any of the following **health facility factors** contribute to the death of this woman? (*lack of facilities, lack of equipment or consumables such as drugs, lack of blood/blood products, lack of theatre supplies/facilities, other, no)* |
| 🡪 if she died, did any of the following **health personnel factors** contribute to the death of this woman? *(*(*lack of human resources, lack of expertise/training or education, poor attitude/low morale, next level cadre not consulted, next level cadre not available, poor interpersonal relationship, language barrier, misdiagnosis/mismanagement, delays in referral, delays in appropriate action, other, no)* |
| 🡪 if she died, did any **other factor(s)** not asked about so far contribute to the death of this woman? *(yes, no)*  If yes, please specify *(textbox)* |
| **Maternal morbidity** |
| **Did the woman have an STI in this pregnancy?** *(yes, no, don’t know)* |
| 🡪 if yes, was the infection due to Chlamydia*? (yes, no, don’t know)* |
| 🡪 if yes, was it treated with tablets or injections? *(yes, no)* |
| 🡪 if yes, was the infection due to Gonorrhoea? *(yes, no, don’t know)* |
| 🡪 if yes, was it treated with tablets or injections? *(yes, no)* |
| 🡪 if yes, was the infection due to Trichomonas? *(yes, no, don’t know)* |
| 🡪 if yes, was it treated with tablets or injections? *(yes, no, don’t know)* |
| 🡪 if yes, was the infection due to Syphilis? *(yes, no, don’t know)* |
| 🡪 if yes, was it treated with tablets or injections? *(yes, no)* |
| 🡪 if yes, was the infection due to other factor(s) not listed above? *(yes, no)* |
| 🡪 if yes, please specify *(text box)* |
| 🡪 if yes, was it treated with tablets or injections? *(yes, no)* |
| 🡪 if yes, was the infection due to an unknown factor? *(yes, no)* |
| **Did the woman have a urinary tract infection in this pregnancy?** *(yes, no, don’t know)* |
| **Did the woman have any other infection in this pregnancy?** *(yes, no, don’t know)* |
| 🡪 if yes, has she had pneumonia? *(yes, no, don’t know)* |
| 🡪 if yes, was she admitted to an intensive care unit (in this facility or elsewhere)? *(yes, no, don’t know)* |
| 🡪 if yes, has she had a kidney infection? *(yes, no, don’t know)* |
| 🡪 if yes, was she admitted to an intensive care unit (in this facility or elsewhere)? *(yes, no, don’t know)* |
| 🡪 if yes, has she had a skin infection? *(yes, no, don’t know)* |
| 🡪 if yes, was she admitted to an intensive care unit (in this facility or elsewhere)? *(yes, no, don’t know)* |
| 🡪 if yes, has she had another type of infection not listed above? *(yes, no, don’t know)* |
| 🡪 if yes, please specify *(text box)* |
| 🡪 if yes, was she admitted to an intensive care unit (in this facility or elsewhere)? *(yes, no, don’t know)* |
| **Potentially life-threatening conditions** |
| **Did the woman have severe malaria?** *(yes, no, don’t know)* |
| 🡪 if yes, what was the timing of the infection relative to birth? *(antepartum, intrapartum, postpartum, don’t know)* |
| **Did the woman have severe systemic infection or sepsis?** (Temp>38, a confirmed/suspected infection, and at least one of: HR>90, RR >20, WCC<4000 or >12,000) *(yes, no)* |
| 🡪 if yes, what was the timing of the infection relative to birth? *(antepartum, intrapartum, postpartum, don’t know)* |
| 🡪 if yes, were antibiotics administered? *(yes, no, don’t know)* |
| 🡪 if yes, was she admitted to an intensive care unit (in this facility or elsewhere) *(yes, no, don’t know)* |
| (**Severe pre-eclampsia with ICU admission**) – assemble definition from components: persistent BP ≥160/110mmHg, proteinuria ≥5g/d, oliguria <400mL in 24hr, HELLP, or pulmonary oedema) |
| **Did the woman have eclampsia?** (generalised fits in a patient without prior epilepsy, includes COMA in pre-eclampsia) *(yes, no)* |
| 🡪 if yes, what was the timing of the 1^st^ seizure relative to birth? *(antepartum, intrapartum, postpartum, don’t know)* |
| (We do not need to ask about severe hypertension.) |
| 🡪 if yes, what was the timing of the condition relative to birth? *(antepartum, intrapartum, postpartum, don’t know)* |
| (We do not need to ask about proteinuria ≥5g/d.) |
| **Did the woman have pulmonary oedema?** *(yes, no)* |
| 🡪 if yes, what was the timing of the condition relative to birth? *(antepartum, intrapartum, postpartum, don’t know)* |
| **Did the woman have a uterine rupture?** *(yes, no)* |
| 🡪 if yes, what was the timing of the condition relative to birth? *(antepartum, intrapartum, don’t know)* |
| **Did the woman have a PPH?** *(yes, no)* |
| 🡪 if yes, did the woman require an intervention with non-pneumatic anti-shock garment *(NASG)? (yes, no)* |
| 🡪 if yes, did the woman require an intervention with balloon tamponade? *(yes, no)* |
| 🡪 if yes, did the woman require an intervention with brace suture? *(yes, no)* |
| 🡪 if yes, did the woman require an intervention with internal iliac artery ligation? *(yes, no)* |
| 🡪 if yes, did the woman require an intervention with hysterectomy? *(yes, no)* |
| 🡪 if yes, did the woman require any other intervention? *(yes, no)* |
| 🡪 if yes, please describe *(text box)* |
| (Abruption is on the delivery screen) |
| **Did the woman have any other potentially life-threatening condition?** *(yes, no) (text box)* |
| 🡪 if yes, what was the timing of the condition relative to birth? *(antepartum, intrapartum, postpartum, don’t know)* |
| **Other complications related to placental disease** |
| **Did the woman have uncontrolled hypertension?** (requiring 3 or more different parenteral agents within a 12hr period) *(yes, no)* |
| 🡪 if yes, what was the timing of the condition relative to birth? *(antepartum, intrapartum, postpartum, don’t know)* |
| **WHO maternal near-miss morbidity*** (excluding those not accepted for SSA use [Tura 2017]) |
| **Clinical criteria** |
| **Did the woman have acute** **cyanosis?** *(yes, no)* |
| 🡪 if yes, what was the timing of the condition relative to birth? *(antepartum, intrapartum, postpartum, don’t know)* |
| **Did the woman have jaundice in the presence of hypertension and ≥1+ proteinuria?** *(yes, no)* |
| 🡪 if yes, what was the timing of the condition relative to birth? *(antepartum, intrapartum, postpartum, don’t know)* |
| **Did the woman suffer a cardiac arrest?** *(yes, no)* |
| 🡪 if yes, what was the timing of the arrest relative to birth? *(antepartum, intrapartum, postpartum, don’t know)* |
| **Did the woman suffer from gasping?** (terminal respiratory pattern in which the breath is convulsively and audibly caught) *(yes, no)* |
| 🡪 if yes, what was the timing of the condition relative to birth? *(antepartum, intrapartum, postpartum, don’t know)* |
| (We do not need to ask about high respiratory rate >27/min (revised definition) or >40/min (original definition), or a RR<6/min? |
| (🡪 if yes, what was the timing relative to birth? *(antepartum, intrapartum, postpartum, don’t know)* |
| (We do not need to ask about shock, defined as sBP <90mmHg or MAP <65mmHg)? |
| (🡪 if yes, did this last for >60 minutes? *(yes, no)* |
| (🡪 If yes, was this despite at least 2L of fluid being administered? *(yes, no)* |
| **Did the woman lose consciousness?** (revised definition) *(yes, no)* |
| 🡪 if yes, did this last for ≥12 hr? *(yes, no) – original definition* |
| **Did the woman suffer any focal neurological sign (revised definition, includes stroke and blindness in setting of hypertension)?** *(yes, no) – original definition included loss of consciousness and stroke, seizure, total paralysis and status/uncontrollable fits* |
| 🡪 if yes, did she suffer a stroke? *(yes, no) - original definition included loss of consciousness and stroke or absence of pulse (cardiac arrest)* |
| 🡪 if yes, did she suffer from blindness? *(yes, no)* |
| 🡪 if yes, what was the timing of the focal neurological sign relative to birth? *(antepartum, intrapartum, postpartum, don’t know)* |
| **Did the woman suffer a generalised seizure lasting >5min, or 2 or more consecutive seizures of any duration with incomplete recovery of consciousness in between?** (revised definition) *(yes, no) – original definition was uncontrollable fit/status epilepticus, total paralysis* |
| 🡪 if yes, what was the timing of the seizure relative to birth? *(antepartum, intrapartum, postpartum, don’t know)* |
| **Did the woman suffer from total paralysis?** *(yes, no)* |
| 🡪 if yes, was her pulse at least 120bpm? *(yes, no)* |
| **Did the woman suffer from oliguria?** *(yes, no) – original definition: oliguria unresponsive to fluids or diuretics (<30ml/hr for 4 hr or <400mL total in 24hr, or revised definition: urine output <0.3ml/kg/hr for ≥24hr or anuria for ≥12hr)* |
| 🡪 if yes, was this <30ml/hr for 4 hr or <400mL total in 24hr? *(yes, no)* |
| 🡪 if yes, was there no urine output for ≥12 hr? *(yes, no)* |
| 🡪 if yes, did this occur despite fluid challenge or diuretics? *(yes, no)* |
| 🡪 if yes, when was the onset relative to birth? *(antepartum, intrapartum, postpartum, don’t know)* |
| **Did the woman suffer from failure to form clots?** (original definition) *(yes, no) – revised is bedside clotting test* |
| 🡪 if yes, what was the timing of the condition relative to birth? *(antepartum, intrapartum, postpartum, don’t know)* |
| **Did the woman suffer from hepatic haematoma or rupture?** *(yes, no)* |
| 🡪 if yes, what was the timing of the condition relative to birth? *(antepartum, intrapartum, postpartum, don’t know)* |
| **Laboratory criteria** |
| (We do not need to ask for lowest oxygen saturation.) |
| **Did the woman’s urine test positive for glucose and ketones** – **diabetic emergency?** *(yes, no, don’t know)* |
| 🡪 if yes, what was the timing relative to birth? *(antepartum, intrapartum, postpartum, don’t know)* |
| **Was the woman’s** **platelet count <50,000?** *(yes, no, not done, don’t know)* |
| 🡪 if yes, what was the timing relative to birth? *(antepartum, intrapartum, postpartum, don’t know)* |
| **Was the woman’s** **peak serum creatinine ≥300mm?** (3.5mg/dL) (original definition) *(yes, no, not done, don’t know)* |
| 🡪 if yes, what was the timing relative to birth? *(antepartum, intrapartum, postpartum, don’t know)* |
| **Did the woman have myocardial ischaemia?** (by ECG changes or markers of myocardial necrosis *(yes, no, not done, don’t know)* |
| 🡪 if yes, what was the timing of the condition relative to birth? *(antepartum, intrapartum, postpartum, don’t know)* |
| **Did the woman suffer hepatic dysfunction or liver failure?** (INR>1.2 in absence of ‘failure to form clots’ or warfarin treatment, in which case, either mixed hyperbilirubinaemia [>1.9mg/dL or >17µM] or hypoglycaemia in absence of insulin [<45mg/dL or <2.5mm]) *(yes, no, not done, don’t know)* |
| 🡪 if yes, what was the timing of the condition relative to birth? *(antepartum, intrapartum, postpartum, don’t know)* |
| **Life-saving management** |
| **Was the woman admitted to ICU?** |
| 🡪 if yes, what was the timing relative to birth? *(antepartum, intrapartum, postpartum, don’t know)* |
| **Was a hysterectomy** **performed for haemorrhage or** **infection?** *(yes, no, don’t know)* |
| **Was the woman transfused with red blood cells?** *(yes, no) – will be used to ascertain whether there was* ***massive transfusion****, defined as ≥5U RBC total or as a revised definition of: ≥10U RBC in 24hr, ≥5U RBC in 1hr with anticipated ongoing need, replacement of 50% of TBV by blood products within 3hr)* |
| 🡪 if yes, how many units did she receive? *(N)* (number box) |
| 🡪 if yes, what was the date and time the first unit was started? *(yyyy/mm/dd and 24:00) (boxes)* |
| 🡪 if yes, what was the date and time the last unit was started? *(yyyy/mm/dd and 24:00 clock)* |
| 🡪 if yes, what was the timing of the first transfusion relative to birth? *(antepartum, intrapartum, postpartum, don’t know)* |
| **Was the woman intubated?** (not related to anaesthesia) *(yes, no)* |
| 🡪 if yes, what was the timing of the intubation relative to birth? *(antepartum, intrapartum, postpartum, don’t know)* |
| **Was cardiopulmonary resuscitation** **performed?** *(yes, no)* |
| 🡪 if yes, what was the timing relative to birth? *(antepartum, intrapartum, postpartum, don’t know)* |
| **Was a laparotomy other than a Caesarean performed?** *(yes, no, don’t know)* |
| **Were parenteral antibiotics administered after delivery?** *(yes, no, don’t know)* |
| **Were anti-malarials administered?** *(yes, no, don’t know)* |
| **Were other parenteral anticonvulsants (e.g., diazepam) administered?** *(yes, no, don’t know)* |
| **Were IV fluids administered?** *(yes, no, don’t know)* |
| **Was a manual removal of the placenta or removal of retained products of conception performed?** *(yes, no, don’t know)* |
| (We do not need to ask about UBT as this is in the PPH question.) |
| **Sample Collection – Women who delivered at a PRECISE facility only** |
| **Was maternal blood collected?** *(yes, no)* |
| **🡪** if no, why? *(no consent, participant refused, participant did not deliver in one of the study sites, no staff member available to collect sample, no staff member available to process the sample, research team were unable to successfully draw blood, participant left before sample was collected, other (specify), don’t know)* |
| **Was maternal urine collected?** *(yes, no)* |
| 🡪 if no, why? *(no consent, participant refused, participant did not deliver in one of the study sites, no staff member available to collect the sample, no staff member available to process the sample, participant unable to produce sample, other (specify), don’t know)* |
| **Was a placental sample collected?** *(yes, no)* |
| 🡪 if no, why? *(No consent, participant refused, placenta discarded prior to collection, participant did not deliver in one of the study sites, no staff member available to collect placenta, no staff member available to process samples, other (specify))* |
| **Was cord blood collected?** *(yes, no)* |
| 🡪 if no, why? *(No consent, participant refused, placenta discarded prior to collection, participant did not deliver in one of the study sites, insufficient blood available, no staff member available to collect the sample, no staff member available to process the sample, other (specify))* |
| **Was an intrapartum vaginal swab collected?** *(yes, no)* |
| 🡪 if no, why? *(no consent, participant refused, participant did not deliver in one of the study sites, no staff member available to collect the sample, no staff member available to process the sample, other (specify), don’t know)* |

**Table S2J: BABY information (for each stillborn and liveborn)**

|  |
| --- |
| **What is the baby’s ID?** (assigned as mother’s ID- # determined by order of birth) |
| **What was the DATE of birth?** *(yyyy/mm/dd or don’t know)* (used to calculate GA at birth) *(date boxes)* |
| **What was the TIME of birth?** *(2400 clock/don’t know)* (used to calculate GA at birth) *(date boxes)* |
| 🡪 if GA<20 weeks, **END OF SCREEN for MISCARRIAGE/TERMINATION** |
| **START OF SCREEN FOR FACILITY-BORN BABIES GA≥20 weeks** |
| **Was the baby born alive?** *(yes, no) (ie. did the baby cry, move or breathe at birth, even a little? – Community question from VA tool)* |
| 🡪 if no, what was the baby’s skin appearance? *(fresh, macerated [peeling or showing signs of decay], don’t know)* |
| 🡪 if no, what was the baby’s sex? *(male, female, indeterminate)* |
| **What was the mode of the delivery?** *(unassisted vaginal without episiotomy, unassisted vaginal with episiotomy, operative vaginal, vaginal breech, resuscitative hysterotomy (perimortem CS), none and the mother died undelivered, caesarean section, don’t know)* (**Facility births only)** |
| 🡪 if no, **FOR BABIES BORN IN FACILITIES ONLY*,** what was the baby’s birthweight? (g) *(enter weight or don’t know (number box or don’t know) – SGA will be calculated using Intergrowth standard*  **this question should be skipped for home births but should be asked of all women who delivered in a facility whether a PRECISE facility or another facility.* |
| 🡪 if no, what was the baby’s length? (cm) *(text box)* |
| 🡪 if no, what was the baby’s mid-upper arm circumference measurement? (cm) *(text box)* |
| 🡪 if no, what was the baby’s head circumference? (cm) *(text box)* |
| 🡪 If no, please provide a case **summary** (short summary of the sequence of events surrounding the death) *(textbox)* |
| 🡪 if no, was an autopsy performed? *(yes, no,* *don’t know)* |
| 🡪 if yes, please provide details *(summary of case [textbox] or unavailable)* |
| 🡪 if yes, what was the perinatal cause of death**? *(congenital malformations, birth trauma, infection, low birth weight, unknown cause, other, don’t know)* |
| 🡪 if no, what was the perinatal cause of death identified clinically**? *(congenital malformations, birth trauma, infection, low birth weight, unknown cause, other, don’t know)* |
| 🡪 if no, did any of the following **personal or family factors** contribute to the death of this baby? *(delay in seeking help, failure to recognise danger signs, declined treatment or admission, ignorance of available services, cultural or religious reasons or objections, poverty, other personal or family factors, don’t know)* |
| 🡪 if no, did factors concerning **access to skilled birth attendance** contribute to the death of this baby? *(yes, no,* *don’t know)* |
| 🡪 if no, did any of the following **transport/communication factors** contribute to the death of this baby? *(lack of transport from home to facility, lack of transport between health facilities, health service communication breakdown, long distance, poor roads, other, no)* |
| 🡪 if no, did any of the following **health facility factors** contribute to the death of this baby? (*lack of facilities, lack or inadequate resuscitation equipment, lack of other equipment or consumables such as drugs (such as (eg. antibiotics, oxygen, anticonvulsants), lack of proper infrastructure (eg. resuscitation area and source of warmth), lack of blood/blood products, lack of proper infection prevention and control practices, lack of theatre supplies/facilities, other, no)* |
| 🡪 if no, did any of the following **health personnel factors** contribute to the death of this baby? *(*(*lack of human resources, lack of expertise/training or education, poor attitude/low morale, next level cadre not consulted, next level cadre not available, poor interpersonal relationship, language barrier, misdiagnosis/mismanagement, delays in referral, delays in appropriate action, other, no)* |
| 🡪 if no, did any **other factor(s)** not asked about so far contribute to the death of this baby? *(yes, no,* *don’t know)*  If so, please specify *(text box)* |
| **END OF SCREEN FOR STILLBORNS** |
| **START OF SCREEN FOR LIVEBIRTHS** |
| **Is the baby still alive?** *(yes, no,* *don’t know)* |
| 🡪 if baby not alive, what was the DATE and TIME of death? *(yyyy/mm/dd and 2400 clock, don’t know)* Please provide as much detail as possible if exact date and time not known ie. how old in days was the baby when he/she died? |
| 🡪 If DK, how old in days was the baby when s/he died? *(numberbox, <24hr, 1-7 d, 8-28 d, >28 d, don’t know)* |
| 🡪 if baby not alive, where did the baby die? *(at hospital, at another health facility, at home, other, don’t know)* |
| 🡪 if baby not alive, what was the neonatal cause of death††? *(congenital malformations, birth trauma, meconium aspiration, infection, low birth weight, prematurity, low birth weight and prematurity, convulsions and disorders of cerebral status, respiratory distress, other cardiorespiratory disorders, unknown cause, other, don’t know)* |
| 🡪 If baby not alive, please provide a case **summary** (short summary of the sequence of events surrounding the death) *(text box)* |
| 🡪 if baby not alive, was an **autopsy** performed? *(yes, no)* |
| 🡪 if yes, please provide details *(summary of case [textbox] or not available)* |
| 🡪 if yes, what was the cause of death? *(congenital malformations, birth trauma, meconium aspiration, infection, low birth weight, prematurity, low birth weight and prematurity, convulsions and disorders of cerebral status, respiratory distress, other cardiorespiratory disorders, unknown cause, other, don’t know)* |
| 🡪 if no, did any of the following **personal or family factors** contribute to the death of this baby? *(delay in seeking help, failure to recognise danger signs, declined treatment or admission, ignorance of available services, cultural or religious reasons or objections, poverty, other personal or family factors, don’t know)* |
| 🡪 if baby not alive, did factors concerning **access to skilled birth attendance** contribute to the death of this baby? *(yes, no,* *don’t know)* |
| 🡪 if no, did any of the following **transport/communication factors** contribute to the death of this baby? *(lack of transport from home to facility, lack of transport between health facilities, health service communication breakdown, long distance, poor roads, other, no)* |
| 🡪 if no, did any of the following **health facility factors** contribute to the death of this baby? (*lack of facilities, lack or inadequate resuscitation equipment, lack of other equipment or consumables such as drugs (such as (eg. antibiotics, oxygen, anticonvulsants), lack of proper infrastructure (eg. resuscitation area and source of warmth), lack of blood/blood products, lack of proper infection prevention and control practices, lack of theatre supplies/facilities, other, no)* |
| 🡪 if no, did any of the following **health personnel factors** contribute to the death of this baby? *(*(*lack of human resources, lack of expertise/training or education, poor attitude/low morale, next level cadre not consulted, next level cadre not available, poor interpersonal relationship, language barrier, misdiagnosis/mismanagement, delays in referral, delays in appropriate action, other, no)* |
| 🡪 if baby not alive, did any **other factor(s)** not asked about so far contribute to the death of this baby? *(yes, no,* *don’t know)*  If so, please specify *(textbox)* |
| **What was the baby’s Apgar score at 1 minute?** *(number box)* |
| **What was the baby’s Apgar score at 5 minutes?** *(number box)* |
| **What is the baby’s sex?** *(male, female, indeterminate)* |
| **Does the baby have any major malformation?** *(yes, no,* *don’t know)* |
| 🡪 if yes, please specify *(swelling/defect on back, very large head, very small head, defect of lip/palate, intestines protruding through the abdomen, other (specify), don’t know)* |
| **Did the baby breathe at birth?** *(yes, no,* *don’t know)* |
| 🡪 if no, was there evidence of meconium? *(yes, no,* *don’t know)* |
| 🡪 if no, what type of resuscitation was used? *(stimulation, bag & mask, both, none, don’t know)* |
| 🡪 if no, who performed resuscitation? *(Paediatrician/Medical Officer/Medical Officer intern/Clinical Officer/Nurse or Midwife/Others (specify))* |
| 🡪 if the baby breathed at birth, did the baby have difficulties breathing at birth? *(yes, no,* *don’t know)* |
| 🡪 if yes, was there evidence of meconium? *(yes, no)* |
| 🡪 if yes, what type of resuscitation was used? *(stimulation, bag & mask, both, don’t know)* |
| 🡪 if yes, was surfactant given? *(yes, no,* *don’t know) – we can compare with GA* |
| **What was the baby’s birthweight?** (g) *(enter weight or don’t know (number box or don’t know)* **(Facility Births only)** |
| **What is the baby’s length?** (cm) *(text box)* **(Facility Births only)** |
| **What is the baby’s head circumference?** (cm) *(text box)* **(Facility Births only)** |
| **What is the baby’s mid-upper arm circumference?** (cm) *(text box)* **(Facility Births only)** |
| **What was the baby’s HR?** (bpm) **(Facility Births only)** |
| **What was the baby’s RR? (**breaths/min based on 30 sec measurement) **(Facility Births only)** |
| **What was the baby’s Hb?** (g/dl) **(Facility Births only)** |
| **What was the baby’s SpO₂?** (%) *(number box)* **(Facility Births only)** |
| **Was the baby admitted to a neonatal ward or unit separate from the mother?** *(yes, no,* *don’t know)* **(Facility Births only)** |
| 🡪 if yes, what was the DATE and TIME of admission? *(yyyy/mm/dd and 2400 or don’t know)* |
| 🡪 if yes, what was the DATE and TIME of discharge? *(yyyy/mm/dd and 2400 or don’t know)* *– if no time given, then assume time was 1200 – duration of neonatal stay will be calculated* *(datebox)* |
| 🡪 if yes, what was the baby’s status at discharge? *(alive/dead/don’t know)* |
| 🡪 if yes, did the baby have fits or seizures during admission? *(yes, no,* *don’t know)* |
| 🡪 if yes, did the baby receive phototherapy for jaundice? *(yes/no, not needed/no, needed but not available/no, needed but declined/don’t know)* |
| 🡪 if yes, did the baby receive oxygen? *(yes/no, not needed/no, needed but not available/no, needed but declined/don’t know)* |
| 🡪 if yes, did the baby receive invasive ventilation [via ETT]? *(yes/no, not needed/no, needed but not available/no, needed but declined/don’t know)* |
| 🡪 if yes, did the baby receive non-invasive ventilation [CPAP]? *(yes/no, not needed/no, needed but not available/no, needed but declined/don’t know)* |
| 🡪 if yes, did the baby receive exchange transfusion for jaundice? *(yes/no, not needed/no, needed but not available/no, needed but declined/don’t know)* |
| 🡪 if yes, did the baby receive antibiotics? *(yes/no, not needed/no, needed but not available/no, needed but declined/don’t know)* |
| 🡪 if yes, was the baby diagnosed with an infection during admission? *(yes, no, don’t know)* |
| 🡪 if yes, what type of infection was the baby diagnosed with? *(tetanus, bacterial meningitis, pneumonia, neonatal sepsis, syphilis, other, don’t know)* |
| 🡪 if other, please specify *(text box)* |
| 🡪 if yes, did the baby receive IV fluids? *(yes/no, not needed/no, needed but not available/no, needed but declined/don’t know)* |
| 🡪 if yes, was a feeding tube used for feeding difficulty or low blood sugar? *(yes/no, not needed/no, needed but not available/no, needed but declined/don’t know)* |
| 🡪 if yes, was the baby put in an incubator for low body temperature? *(yes/no, not needed/no, needed but not available/no, needed but declined/don’t know)* |
| **Were the baby’s nose or mouth suctioned?** *(yes, no, don’t know)* **(Facility Births only)** |
| **Was the baby breastfed or fed breast milk?** *(yes, no, don’t know)* **(Facility Births only)** |
| 🡪 if yes, how long after birth did the baby first breastfeed or receive breast milk? *(<1 hour, <2 hours, <4 hours, <12 hours, >12 hours (please specify))* |
| **What is the baby’s breastfeeding status at discharge?** *(feeding ok/is not breastfeeding or has troubles/don’t know)* **(Facility Births only)** |
| **Did the baby receive kangaroo mother care?** *(yes, no, don’t know)* **(Facility Births only)** |
| **Was the baby bathed?** *(yes, no, don’t know)* **(Facility Births only)** |
| 🡪 if yes, how long after birth was the baby bathed? *(<1 hour, 1<2 hours, 2<4 hours, 4<12 hours, =>12 hours (please specify))* |
| **Did the baby receive Vitamin K?** *(unavailable, yes, no, don’t know)* **(Facility Births only)** |
| **Is the mother HIV positive?** *(yes, no, don’t know)* **(Facility Births only)** |
| 🡪 if yes, was ART for infant prophylaxis initiated? *(no/yes at diagnosis/yes only for labour and delivery, don’t know)* |
| **Sample Collection – Babies who were born at a PRECISE facility in The Gambia and Mozambique only** |
| **Was neonatal heel prick collected?** *(yes, no)* |
| 🡪 if no, why? *(no consent, participant refused, no staff member available to collect the sample, no staff member available to process the sample, not needed – cord blood sample collected, other (specify), don’t know)* |

**Table S2K: Woman’s Discharge (all live women, to be completed at the time they leave the facility)**

| **When after birth did the woman leave the facility?** *(yyyy/mm/dd, hh/mm, don’t know)* |
| --- |
| **Where did the woman go when she left this facility?** *(Own home, relative’s home, friend’s home, transferred to another facility, other (specify), don’t know)* |
| 🡪If the woman was transferred to another facility, which facility was she transferred to? (*Text box)* |
| 🡪If the woman went home, was this against medical advice? *(yes, no, don’t know)* |
| 🡪If the woman went home, was she prescribed any therapy? *(yes, no, don’t know)* |
| 🡪If yes, which therapy was she prescribed? (*Text box)* |
| 🡪If the woman went home, was she advised to return sooner than routine? *(yes, no, don’t know)* |
| 🡪If yes, after how many days was she advised to return? *(Number box)* |
| 🡪If the woman went home, was she referred to another service within this facility for specialist follow-up? *(yes, no, don’t know)* |
| 🡪If yes, please specify which other service she was referred to? *(Text box)* |
| 🡪If the woman went home was she referred to another service not in this facility for specialist follow-up? *(yes, no, don’t know)* |
| 🡪If yes, please specify which other service she was referred to? *(Text box)* |

**Table S2L: Infant(s) Discharge (all live babies, to be completed at the time they leave the facility)**

| **When after birth did the baby leave the facility?** *(yyyy/mm/dd, hh/mm, don’t know)* |
| --- |
| **Where did the baby go when he/she left this facility?** *(Mother’s home, relative’s home, family friend’s home, transferred to another facility, other (specify), don’t know)* |
| 🡪If the baby was transferred to another facility, which facility was (s)he transferred to? *(Textbox)* |
| 🡪If the baby went home, was this against medical advice? *(yes, no, don’t know)* |
| 🡪If the baby went home, was (s)he prescribed any therapy? *(yes, no, don’t know)* |
| 🡪If yes, which therapy was (s)he prescribed? *(Textbox)* |
| 🡪If the baby went home, was the family advised to bring the baby back sooner than routine? *(yes, no, don’t know)* |
| 🡪If yes, after how many days were they advised to bring the baby back? *(Number box)* |
| 🡪If the baby went home, was the baby referred to another service within this facility for specialist follow-up? *(yes, no, don’t know)* |
| 🡪If yes, please specify which other service (s)he was referred to? *(Text box)* |
| 🡪If the baby went home, was the baby referred to another service not in this facility for specialist follow-up? *(yes, no, don’t know)* |
| 🡪If the baby went home, was the family given any particular advice? *(yes, no, don’t know)* |

**Table S2M: Laboratory**

| **Laboratory Tests** |
| --- |
| **Laboratory Test Date** *(yyyy/mm/dd)* |
| **Screened for GDM?** (*done and result available, done and result not yet available, not done)* |
| 🡪 If yes, what was the date of screening? *(yyyy/mm/dd)* |
| 🡪 if yes, what type of test was performed? *(1-Fasting blood glucose (ID=1), 2-Glucose challenge test (ID=2), 3-Glucose tolerance test (ID=3)) –* ***ADD*** *Glycosuria by urinary dipstick, random blood glucose)* |
| 🡪 if glucose challenge or tolerance tests were performed, what was her fasting glucose level? *(mmol/L, mg/dL,* *unavailable*) *(from textbox)* |
| 🡪 if glucose challenge or tolerance tests were performed, what was her 1h glucose level? *(mmol/L, mg/dL,* *unavailable*) *(from textbox)* |
| 🡪 if glucose challenge or tolerance tests were performed, what was her 2h glucose level? *(mmol/L, mg/dL,* *unavailable*) *(from textbox)* |
| 🡪 if glucose challenge or tolerance tests were performed, what was her 3h glucose level? *(mmol/L, mg/dL, unavailable)* *(from textbox)* |
| 🡪 if random blood sugar test was performed, what was the result? *(mmol/L, mg/dL, unavailable)* |
| 🡪 if glycosuria testing was performed, what was the result? *(None, 1+, 2+, 3+)* |
| **Haemoglobin level tested?** *(done and result available, done and result not yet available, not done)* |
| 🡪 if done and result available, haemoglobin result *(g/L, g/dL)* |
| **Haematocrit tested?** *(done and result available, done and result not yet available, not done)* |
| 🡪 if done and result available, haematocrit result *(%)* |
| **White Cell Count tested?** *(done and result available, done and result not yet available, not done)* |
| 🡪 if done and result available, White Cell Count result *(N cells x 10^9/L)* |
| **Platelet Count tested?** *(done and result available, done and result not yet available, not done)* |
| 🡪 if done and result available, Platelet Count result *(N cells x 10^9/L)* |
| **Serum Creatinine tested?** *(done and result available, done and result not yet available, not done)* |
| 🡪 if done and result available, Serum Creatinine result *(µM or mEq/L)* |
| **Aspartate transaminase tested?** *(done and result available, done and result not yet available, not done)* |
| 🡪 if done and result available, Aspartate Transaminase result *(IU/L)* |
| **Alanine aminotransferase tested?** *(done and result available, done and result not yet available, not done)* |
| 🡪 if done and result available, Alanine Aminotransferase result *(IU/L)* |
| **Rhesus status tested?** *(done and result available, done and result not yet available, not done)* |
| 🡪 if done and result available, Rhesus status result *(negative, positive)* |
| **HIV tested?** *(done and result available, done and result not yet available, not done)* |
| 🡪 if done and result available, HIV Screening result *(negative, positive, inconclusive)* |
| **CD4 count tested?** *(done and result available, done and result not yet available, not done)* |
| 🡪 if done and result available, CD4 Count result *(cells/mm^3)* |
| **Viral load tested?** *(done and result available, done and result not yet available, not done)* |
| 🡪 if done and result available, Viral Load result *(copies/mL)* |
| **TB tested?** *(done and result available, done and result not yet available, not done)* |
| 🡪 if done and result available, TB result *(negative, positive)* |
| **Malaria tested?** *(done and result available, done and result not yet available, not done)* |
| 🡪 if done and result available, Malaria result *(negative, positive)* |
| **Syphilis VDRL tested?** *(done and result available, done and result not yet available, not done)* |
| 🡪 if done and result available, VDRL test result *(reactive, non-reactive)* |
| **Syphilis RDT tested?** *(done and result available, done and result not yet available, not done)* |
| 🡪 if done and result available, RDT test result *(reactive, non-reactive)* |
| **Syphilis RPR tested?** *(done and result available, done and result not yet available, not done)* |
| 🡪 if done and result available, RPR test result *(reactive, non-reactive)* |
| **Stool tested for ova and parasites?** *(done and result available, done and result not yet available, not done)* |
| 🡪 if done and result available, stool test for ova and parasites result *(identified: hookworm, nothing identified, other)* |
| **Other investigation(s) carried out (1)?** *(done and result available, done and result not yet available, not done)* |
| 🡪 if done and result available, other test result *(textbox)* |
| 🡪 if done and result available, other test result unit *(textbox)* |
| **Other investigation(s) carried out (2)?** *(done and result available, done and result not yet available, not done)* |
| 🡪 if done and result available, other test result *(textbox)* |
| 🡪 if done and result available, other test result unit *(textbox)* |
| **Other investigation(s) carried out (3)?** *(done and result available, done and result not yet available, not done)* |
| 🡪 if done and result available, other test result *(textbox)* |
| 🡪 if done and result available, other test result unit *(textbox)* |
| **Other investigation(s) carried out (4)?** *(done and result available, done and result not yet available, not done)* |
| 🡪 if done and result available, other test result *(textbox)* |
| 🡪 if done and result available, other test result unit *(textbox)* |
| **Other investigation(s) carried out (5)?** *(done and result available, done and result not yet available, not done)* |
| 🡪 if done and result available, other test result *(textbox)* |
| 🡪 if done and result available, other test result unit *(textbox)* |

**Table S2N: Withdrawal/Lost to follow-up**

| **Did the woman withdraw from the study?** *(No, yes)* |
| --- |
| 🡪 if yes, please state the major reason for withdrawal *(unhappy with the study, no reason given,*  *other (please specify)* |
| 🡪 if yes, does the woman give consent to have her and her baby’s information collected from  medical records? *(No, yes)* |
| **Was the woman lost to follow-up?** *(No, yes)* |
| 🡪 if yes, please specify the date on which this decision was made *(yyyy/mm/dd)* |
